# Emergence of neutralizing RBD antibodies following Omicron infection with limited activity against ancestral SARS-CoV-2

**DOI:** 10.1016/j.isci.2026.117166

**Published:** 2026-08-11

**Authors:** Michael S. Piepenbrink, Yao Ma, Simran Panjwani, Alexandra R. Blake, Allison M. Bell, James L. Kizziah, Sara H. Mahmoud, Gregory C. Ippolito, Nathan B. Erdmann, Paul A. Goepfert, Luis Martinez-Sobrido, James J. Kobie, Mark R. Walter

**Affiliations:** 1 Heersink School of Medicine, Infectious Diseases Division, University of Alabama at Birmingham, Birmingham, AL 35294, USA; 2 Department of Disease Intervention and Prevention, Texas Biomedical Research Institute, San Antonio, TX 78227, USA; 3 Heersink School of Medicine, Department of Microbiology, University of Alabama at Birmingham, Birmingham, AL 35294, USA

**Keywords:** SARS-CoV-2, Omicron, variant, KP3.1.1, broadly neutralizing antibody, cryoEM, structure

## Abstract

Severe acute respiratory syndrome coronavirus 2 (SARS-CoV-2) evolution reduces the efficacy of prophylactic vaccines and monoclonal antibody (mAb) therapies. To evaluate potency and breadth, receptor binding domain (RBD)-targeting neutralizing mAbs were identified from patient B cells using an Omicron KP3.1.1 Spike (S) protein bait. MAbs exhibiting the greatest neutralization potency (1332D4 and 1332E5) and binding breadth (1324A10 and 1316C10) were evaluated in greater detail. Cryo-electron microscopy (Cryo-EM) studies revealed 1332D4 and 1332E5 target RBD residue segments 439–446 and 498–506 of KP3.1.1 S, respectively. 1332E5 is a knob^498-506^-targeting class-1/4 mAb that exhibits pan-Omicron specificity but does not bind or neutralize ancestral Wuhan-1. Further analysis suggests mAbs generically defined as class 1/4 mAbs may be separated into two classes. Class 1/4 mAbs, like 1332E5, and class 4/1 mAbs that make extensive interactions with the class-4 epitope and limited contacts with the class-1 knob^498-596^. Despite these differences, the specificity of both mAb classes can be altered by mutations in knob^498-506^.

## Highlights

• SARS-CoV-2 neutralizing antibodies identified from B-cells
• Omicron specific and broadly neutralizing mAbs characterized
• Structure analysis distinguishes class 1/4 and class 4/1 mAbs
• Shared eptiope of class 1/4 and 4/1 mAbs are RBD residues 498-506

## Graphical abstract

**Figure:**
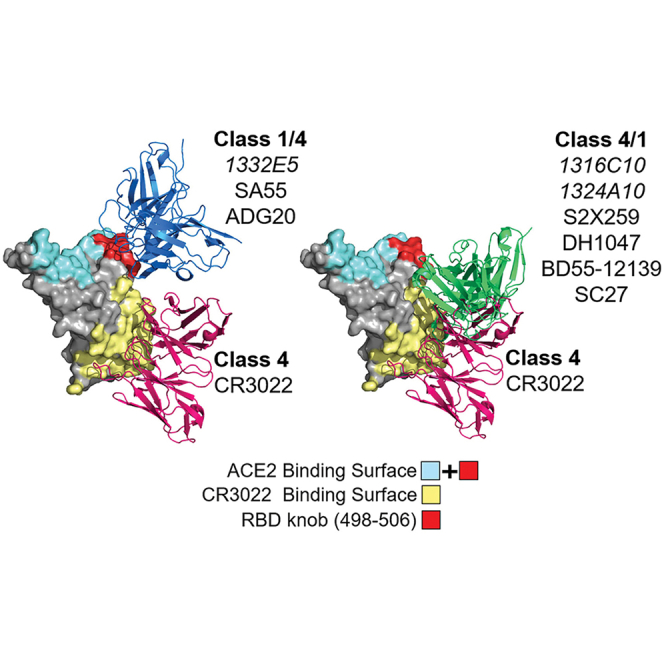

## Teaser

Natural sciences; Biological sciences; Immunology; Microbiology; Virology

## Introduction

Severe acute respiratory syndrome coronavirus 2 (SARS-CoV-2) continues to evolve since its emergence in Wuhan, Hubei Province, China in December 2019. Through a combination of immunity attributed to vaccination or previous infection, and viral mutations resulting in less virulence, the severity of symptoms and morbidity has greatly declined since the peak of the COVID-19 pandemic. Deaths in the United States attributed to SARS-CoV-2 infection have declined from ∼385,000 in 2020 to ∼47,000 in 2024, and only ∼20,000 in 2025.^1^ The use of anti-virals such as nirmatrelvir/ritonavir and remdesivir have continued to contribute to reductions in SARS-CoV-2 mortality.^2^^,^^3^^,^^4^ However, they have limitations that include drug-drug interaction considerations, and viral rebound after treatment.^5^ The clinical use of monoclonal antibodies (mAbs) for treatment of SARS-CoV-2 infection has been less prevalent, at least in part due to cost and dosing considerations, and in more recent years a reduction in neutralizing activity against emerging variants. However clinical use of mAbs may enable treatment of individuals not eligible for treatment with other anti-virals, have potential as an adjunctive therapy, or be an alternative for prophylaxis.

The receptor binding domain (RBD) of the SARS-CoV-2 spike (S) protein consistently remains the target of the most potent neutralizing antibodies.^6^ This is largely due to the increased potency of RBD-targeting antibodies, relative to other S epitopes, that directly inhibit RBD binding to ACE2 on host cells and thus prevent viral attachment. However, since the initial emergence of ancestral SARS-CoV-2 Wuhan-1 strain, the RBD has undergone antigen drifts and shifts resulting in the emergence of new variants that often disrupt the activity of therapeutic or vaccine-induced RBD antibodies.^7^^,^^8^ Consequently, currently there are no longer any U.S. Food and Drug Administration (FDA)-approved mAbs for treatment of SARS-CoV-2 infection, and few remain approved for prophylactic use.^9^ This vividly articulates the continuing co-evolution of SARS-CoV-2 and the human antibody response against it.

The Omicron variant first emerged in November 2021 and has dominated infections in the years following.^8^^,^^10^ There have been many sub-variants of Omicron from the early BA.1 and BA.2 to the later XBB.1.5 and the divergent BA.2.86/JN.1 lineages that include KP.1, LP8.1 and XFG (a hybrid of LF.7 and LP8.1.2).^11^^,^^12^ This continuing diversification necessitates ongoing mechanistic evaluation of the human humoral response to SARS-CoV-2 with attention to the breadth and potency of neutralizing antibodies in context of contemporary dominant S proteins. Here we provide the mechanistic and structural basis of RBD neutralizing antibodies isolated from convalescent individuals when JN.1 and KP.3 infections were at their peak. Notably, two RBD antibodies (1332D4 and 1332E5) exhibited high neutralization potency against current SARS-CoV-2 variants, but lost the ability to bind ancestral Wuhan-1 S. One mAb (1332D4) predominantly targets RBD position 445, consistent with immune pressure contributing to the V445R mutation found in the XFG variant. The other mAb (1332E5) exhibits potent and broad neutralizing activity against Omicron variants, shares the same epitope used by the ADG20/pemivibart, and protects mice from lethal Omicron infection. Analysis of 1332E5 distinguishes two distinct mAb classes. Class 1/4 mAbs that form extensive contacts with RBD knob^498-506^ and minor RBD class-4 epitope contacts and class 4/1 mAbs that make extensive RBD class 4 epitope contacts and minimal interactions with the RBD knob^498-506^. Sequence conservation of the RBD knob^498-506^ is evaluated to extend our understanding of the unique specificities of these mAb classes.

## Results

### Isolation and molecular features of SARS-CoV-2 RBD mAbs following Omicron infection

**Figure 1:**
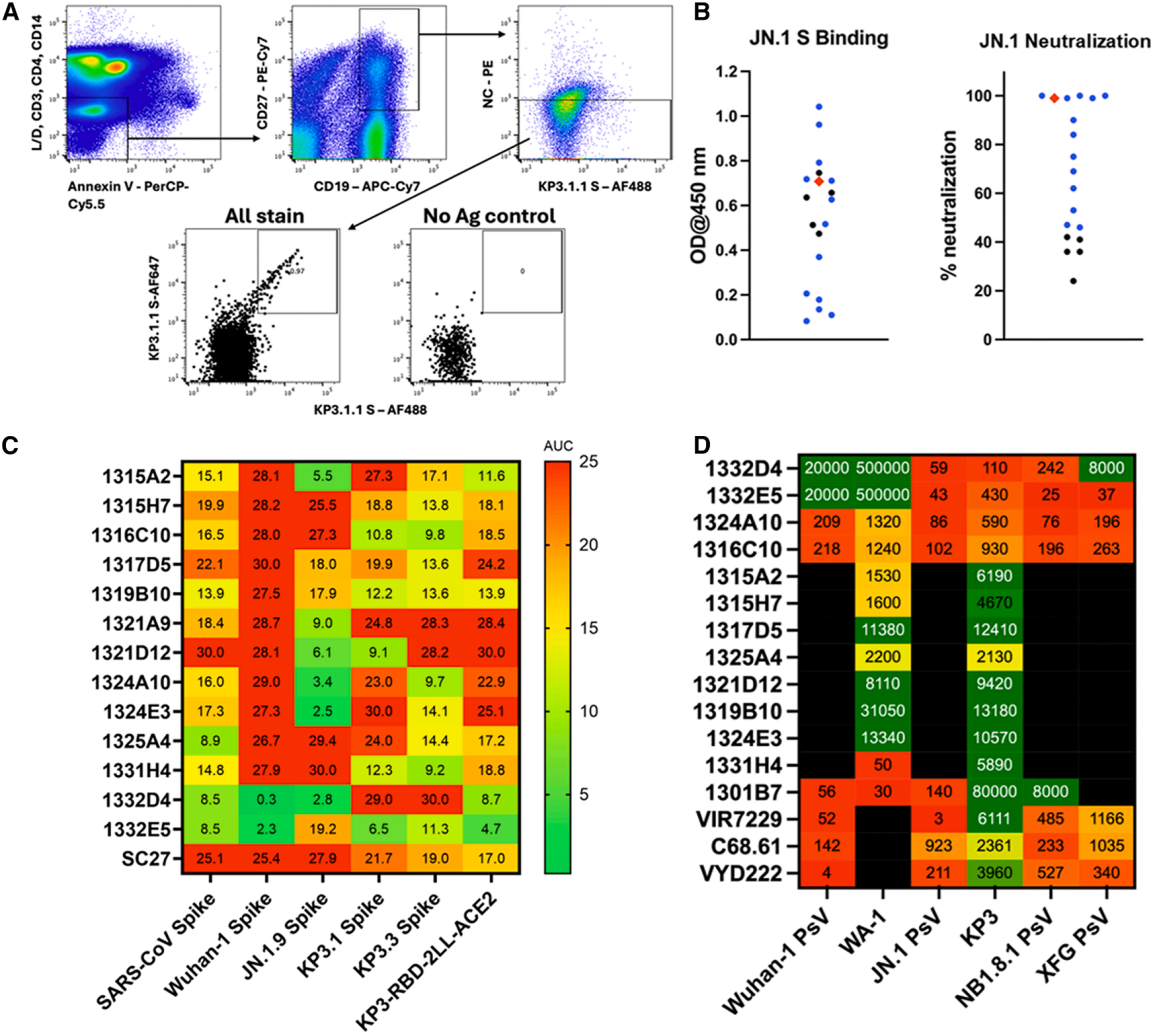
Isolation and screening of SARS-CoV-2 S mAbs (A) Representative strategy for isolation of SARS-CoV-2-specific B cells by flow cytometry. NC-PE corresponds to PE-labeled SARS-CoV-2 nucleocapsid protein. (B) Screening of mAbs for binding to JN.1 S tested at 10 μg/mL by ELISA and neutralization of JN.1 pseudovirus tested at 2 μg/mL. Red diamond indicates SC27 comparator mAb. Blue dots represent 13 mAbs downselected for further analysis. (C) ELISA mAb binding screen with indicated spike proteins. The ELISA was performed with 1 and 10 μg/mL of antibody in triplicate. The OD 450 values were reduced to area under the curve values. A normalization adjustment for each spike dataset was made so that the highest value in each dataset was divided by 30 and then each value in the dataset was divided by the calculated quotient. This correction allows for a clearer comparison in the heatmap that was generated. (D) NT50 values (ng/mL) derived from microneutralization of live SARS-CoV-2 virus using the WA.1 and KP.3 in the presence of 200 pfu/well, or pseudotyped virus containing the spike protein of the Wuhan-1, JN.1, NB1.8.1 or XFG.

To provide insight into RBD antibody responses, peripheral blood memory B cells from individuals who had SARS-CoV-2 infection between August 2023 and August 2024 were isolated. During this period, JN.1 and KP.3 were the dominant variants in circulation. Thus, JN.1 and KP.3.1.1 pre-fusion S protein baits were used to isolate B cells for mAb generation (Figure 1A). A total of 18 mAbs were isolated that exhibited some degree of binding to SARS-CoV-2 JN.1 S and neutralization of SARS-CoV-2 JN.1 pseudovirus (PsV) during screening (Figure 1B). Five mAbs exhibited poor JN.1 neutralization (<45% at 2 μg/mL) and were excluded from further analysis. The remaining 13 mAbs exhibited diverse VDJ usage (Table 1) with three using IGHV3-30, three using IGHV5-51, with the remaining seven using various other variable heavy genes. The HCDR3 ranged in length from 10 to 21 amino acids and a mean VH amino acid mutation rate of 12.3% (±4.1) of heavy variable chains. For the light chain, IGLV6-57 was used five times, and IGKV1-5 and IGKV1-39 both used twice. Amino acid mutations of the variable light chain averaged 9.1% (±3.1).

**Table 1:** Monoclonal antibody characteristics

mAb ID	Chain	V	D	J	Mutations %nt/%AA	CDR3	Isotype
1315A2	heavy	IGHV3-30∗02	IGHD4-23∗01	IGHJ4∗02	7.8/16.3	AKVEWLRGSFDH	IgG1
	light	IGLV6-57∗01		IGLJ3∗02	4.7/9.2	QSYDSTDHWV	
1315H7	heavy	IGHV3-23∗04	IGHD6-19∗01	IGHJ5∗01	5.8/8.6	ARPRTGWYVGIDY	IgG1
	light	IGLV6-57∗02		IGLJ3∗02	4.4/9.2	QSFDSTSLWV	
1316C10	heavy	IGHV5-51∗01	IGHD3-10∗02	IGHJ4∗02	8.5/20.4	VRLLEAPMIKLFAYFDS	IgG1
	light	IGLV6-57∗03		IGLJ3∗02	3.7/6.1	QSYDGNHLWV	
1317D5	heavy	IGHV3-33∗01	IGHD4-17∗01	IGHJ5∗01	5.4/10.2	ARDQGTTVTWFDS	IgG1
	light	IGKV1-39∗01		IGKJ1∗01	5.2/9.5	QQSYNTPPWT	
1319B10	heavy	IGHV7-4-1∗02	IGHD4-17∗01	IGHJ5∗02	5.4/9.2	AREYGDYIISFDP	IgG1
	light	IGKV6-21∗01		IGKJ2∗01	2.5/5.3	HQSSSLPYT	
1321A9	heavy	IGHV5-51∗01	IGHD1-7∗01	IGHJ4∗02	7.1/11.2	AGSGTTGYFEY	IgG1
	light	IGKV1-5∗01		IGKJ2∗03	2.8/6.3	QQYNSNSPYT	
1321D12	heavy	IGHV3-30∗04	IGHD3-22∗01	IGHJ4∗02	8.6/16.5	AMSPGPYYYDNGGYWPSGFDY	IgG^U^
	light	IGKV1-5∗01		IGKJ2∗03	6.0/11.7	QQYNTYHS	
1324A10	heavy	IGHV4-59∗01	IGHD3-3∗01	IGHJ5∗01	7.9/15.5	ARGRGVFGVITLIDT	IgG1
	light	IGLV6-57∗02		IGLJ3∗02	6.8/10.3	LSYHSGNWV	
1324E3	heavy	IGHV3-30∗01	IGHD6-13∗01	IGHJ4∗02	6.4/12.2	ARDGDGRTYYFDS	IgG1
	light	IGKV1-39∗01		IGKJ5∗01	7.4/14.9	QQSYSTLAIT	
1325A4	heavy	IGHV3-53∗04	IGHD1-1∗01	IGHJ4∗02	6.5/10.3	ARHDTWPRGQLDY	IgA1
	light	IGLV6-57∗02		IGLJ2∗01	4.1/6.1	QSYDSSNHVV	
1331H4	heavy	IGHV3-66∗01	IGHD6-13∗01	IGHJ4∗02	11.2/14.4	ARDIPAEDTL	IgG1
	light	IGKV1-33∗01		IGKJ4∗01	7.7/14.7	HQYDNLPRT	
1332D4	heavy	IGHV5-51∗01	IGHD5-24∗01	IGHJ5∗02	5.8/10.2	VRPDGVNDWFGVDP	IgG2
	light	IGKV3-15∗01		IGKJ2∗01	5.6/8.4	QQYNHWPPYT	
1332E5	heavy	IGHV4-61∗12	IGHD2-15∗01	IGHJ3∗02	4.5/5.2	ATYVRLGGFWSGEADAFDI	IgG2
	light	IGLV2-23∗01		IGLJ1∗01	3.7/7.1	CLYADSTTYV	

^U^Sub-class cannot be identified except that it is not IgG1.

### Characterization of mAb binding specificity and epitopes

The binding profile of the candidate mAbs were evaluated by ELISA against pre-fusion S proteins from SARS-CoV, and SARS-CoV-2 Wuhan-1, JN.1.9, KP3.1.1, and KP3.3 (Figure 1C). The previously described broadly neutralizing RBD mAb, SC27^13^ was included as a comparator. To potentially identify mAbs that bind highly conserved residues within the ACE2 receptor binding motif (RBM) of RBD, mAb binding to a RBD^KP3^-ACE2 fusion protein (KP3-RBD-2LL-ACE2) was also characterized. The RBD-ACE2 complex formed by KP3-RBD-2LL-ACE2 should block mAbs with specificity for the RBM, providing a strategy to differentially profile RBM-specific mAbs.^14^ Theoretically, an RBM-specific mAb would have low binding to the KP3-RBD-2LL-ACE2 fusion protein but higher binding to uncomplexed RBD or S.

**Figure 2:**
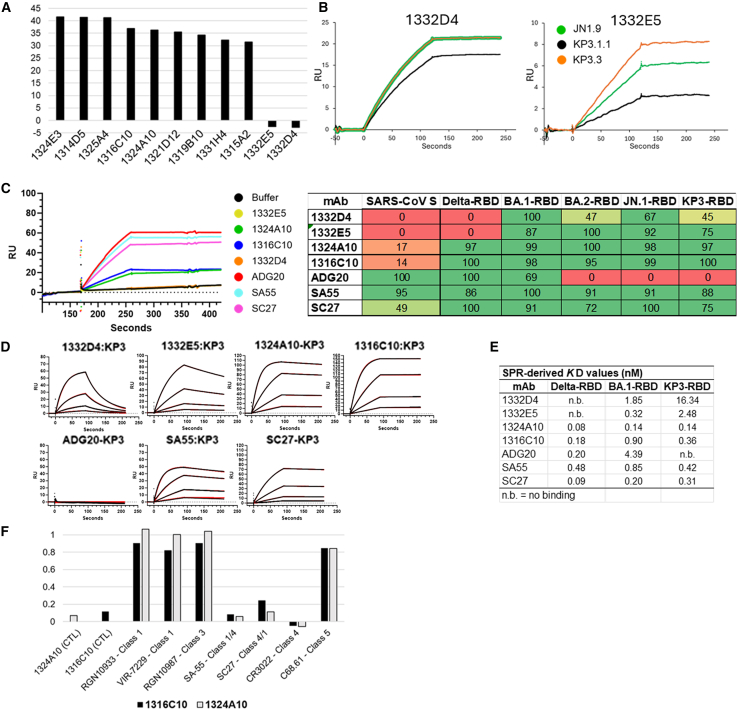
Evaluation of mAb binding (A) SPR response units (RU) were recorded after injection of 20 nM mAb over Wuhan-1 RBD-Fc that was captured on a CM5 Series S sensor chip. The experiment was repeated at least 3 times for each mAb to establish a standard deviation of ± 0.6RU for each experiment. (B) Single injection (20 nM) binding profiles were defined for 1332D4 and 1332E5 binding to JN1.9, KP3.1.1, and KP3.3 S trimers captured to an NTA chip surface. Equivalent levels of S trimers were captured for all experiments. (C) Specificity studies evaluate mAb binding to SARS-CoV S (raw sensorgrams shown) and several variant RBDs. For these studies, mAbs were captured to the chip surface followed by injection of 20 nM S or RBD. The data for all experiments are summarized in the table, where the greatest binding response for each mAb was used to normalize responses. SPR sensorgrams for mAbs binding to RBD^KP3^ (D) and *K*D values for mAbs binding to SARS-CoV-2 RBDs from Delta, BA.1, and KP3. All sensorgrams and binding rate constants are reported in Figure S3. (E) SPR-derived equilibrium dissociation constants (*K*D) for the mAbs binding to different RBDs. (F) Epitope mapping of 1324A10 and 1316C10 by SPR competition assay. 132410 or 1326C10 (50 nM) was injected over RBD^Wuhan-1^-Fc, followed by a 50 nM injection of a second mAb as labeled in the figure. Values reported are the fractional RU responses of the mAb in the presence of 1316C10 or 1324A10 divided by the RU response of a 50 nM injection of the mAb without 1316C10 or 1324A10. 1316C10 CTL and 1324A10 CTL values are for double injections of these mAbs. e.g., evaluating their ability to block their own epitope.

ELISA analysis confirmed all mAbs bound JN.1.9, KP3.1.1, and KP.3.3 S, but there was considerable binding variability, particularly to JN.1.9 (Figure 1C). Most striking was the loss of binding to the original Wuhan-1 S for two of the mAbs, 1332D4 and 1332E5. These two mAbs also exhibited the poorest binding to the KP3-RBD-2LL-ACE2 fusion protein. To corroborate these findings, mAb binding to an RBD^Wuhan-1^-Fc fusion protein was determined by surface plasmon resonance (SPR) (Figure 2A). The assay confirmed all mAbs bound to the Wuhan-1 RBD, except 1332D4 and 1332E5, extending the ELISA analysis. Epitope overlap between the mAbs was evaluated by SPR (Figure S1). All mAbs exhibited overlapping epitopes except 1321D12 and 1331H4 that exhibited distinct non-overlapping epitopes with each other and the group of mAbs with overlapping epitopes. To further study the unique 1332D4 and 1332E5 binding properties and address the general variability of mAb binding in the ELISA data, SPR studies were performed with JN1.9, KP3.1.1, and KP3.3 S proteins attached to an NTA SPR chip through their C-terminal his-tags (Figure 2B). The SPR analysis exhibited the same binding variability observed in the ELISA. For example, initial studies found 1332D4 and 1332E5 both bound strongly to JN1.9. However, repeating the analysis resulted in robust 1332D4 binding but limited binding of 1332E5. Analysis of the sensorgrams confirmed low binding levels for 1332E5, but a very slow dissociation rate for the interaction (Figure 2B), suggesting that binding variability was due to alteration in accessibility of the binding epitopes and not poor binding of either 1332D4 or 1332E5 to S variants.

The neutralizing profile of the mAbs was determined against authentic ancestral WA.1 and KP.3 SARS-CoV-2 strains (Figures 1D and S2). All mAbs neutralized KP.3 with NT_50_ values ranging from 110 to 13180 ng/mL. Consistent with their binding profiles, all mAbs neutralized ancestral WA.1, except 1332D4 and 1332E5. Notably, 1332D4 (NT_50_ = 110 ng/mL) and 1332E5 (NT_50_ = 430 ng/mL) exhibited the most potent neutralization against KP.3. Because 1332D4 and 1332E5 exhibit extremely diverse NT_50_ for WA.1 and KP.3 (e.g., essentially none for ancestral WA.1 and potent neutralization against KP.3), NT_50_ values were compared for the other 10 mAbs by assigning WA.1/KP.3 NT_50_ ratios within 30% of each other as having equivalent potency for both viruses. Excluding 1332D4 and 1332E5, five mAbs exhibited equivalent potency for both WA.1 and KP.3 (1316C10, 1317D5, 1325A4, 1321D12, 1324E3), three mAbs (WA.1/KP.3 = 0.009–0.34) had reduced potency for KP.3 (1315A2, 1315H7, 1331H4), and 2 mAbs (WA.1/KP.3 = 2.2–2.4) exhibited higher potency for KP.3, relative to WA.1 (1324A10, 1319B10). Overall, the mAbs followed the general trend that the most potent mAbs to ancestral WA.1 strain exhibited reduced potency to the divergent variant.

Four mAbs (1332D4, 1332E5, 1324A10, and 1316C10) exhibiting the highest potency for KP.3 were screened for their ability to bind SARS-CoV and other Omicron variants (Figures 2C and S1B), as well as neutralize PsV-expressing S from Wuhan-1, JN.1.9, and newer evolving SARS-CoV-2 Omicron strains NB.1.8.1 and XFG (Figure 1D). The data show 1332D4 and 1332E5 are Omicron-specific mAbs, while 1324A10 and 1316C10 exhibit broad specificity that includes affinity for SARS-CoV. PsV assays show 1332D4 and 1332E5 failed to neutralize ancestral Wuhan-1 virus, but potently neutralized JN.1.9 and NB1.1.8.1, while 1332E5 retained ability to potently neutralize all Omicron variants tested, including the recent XFG variant (NT_50_ = 37 ng/mL). 1316C10 and 1324A10, consistent with their pan-SARS-CoV/-2 binding and neutralizing profiles exhibited activity against Wuhan-1, JN.1.9, NB.1.8.1, and XFG, although with less potency against Omicron variants as compared to 1332E5. 1332E5 exhibited superior neutralization potency against NB.1.8.1 and XFG variants compared to 1332D4, 1316C10, and 1324A10. 1332E5, 1316C10, and 1324A10 all exhibited greater potency than previously described mAbs, VIR7229, C68.61, and VYD222.^15^^,^^16^^,^^17^ Our results are consistent with past studies that have shown VYD222 has reduced activity against later Omicron variants.^18^^,^^19^^,^^20^

The binding affinity of 1324A10, 1316C10, 1332D4, 1332E5, and comparator mAbs (ADG20, SA55, and SC27), for RBD domains from SARS-CoV-2 Delta, BA.1 and KP.3 variants, were evaluated by SPR (Figures 2D, 2E, and S3). 1324A10 and 1316C10 exhibited *K*D values less than 1 nM for all three RBDs evaluated. Despite being more potent in neutralization assays (Figure 1D), 1332D4 and 1332E5 exhibited significantly lower binding affinity for KP.3 than 1324A10 and 1316C10 (Figure 2E). As discussed later in discussion, this is likely due to differences in epitope accessibility for 1332E5, while the complete 1332D4 binding epitope consists of two RBDs and requires a trimeric S for high affinity binding.

### Effectiveness of mAbs in protecting K18 hACE2 transgenic mice following lethal viral challenge

**Figure 3:**
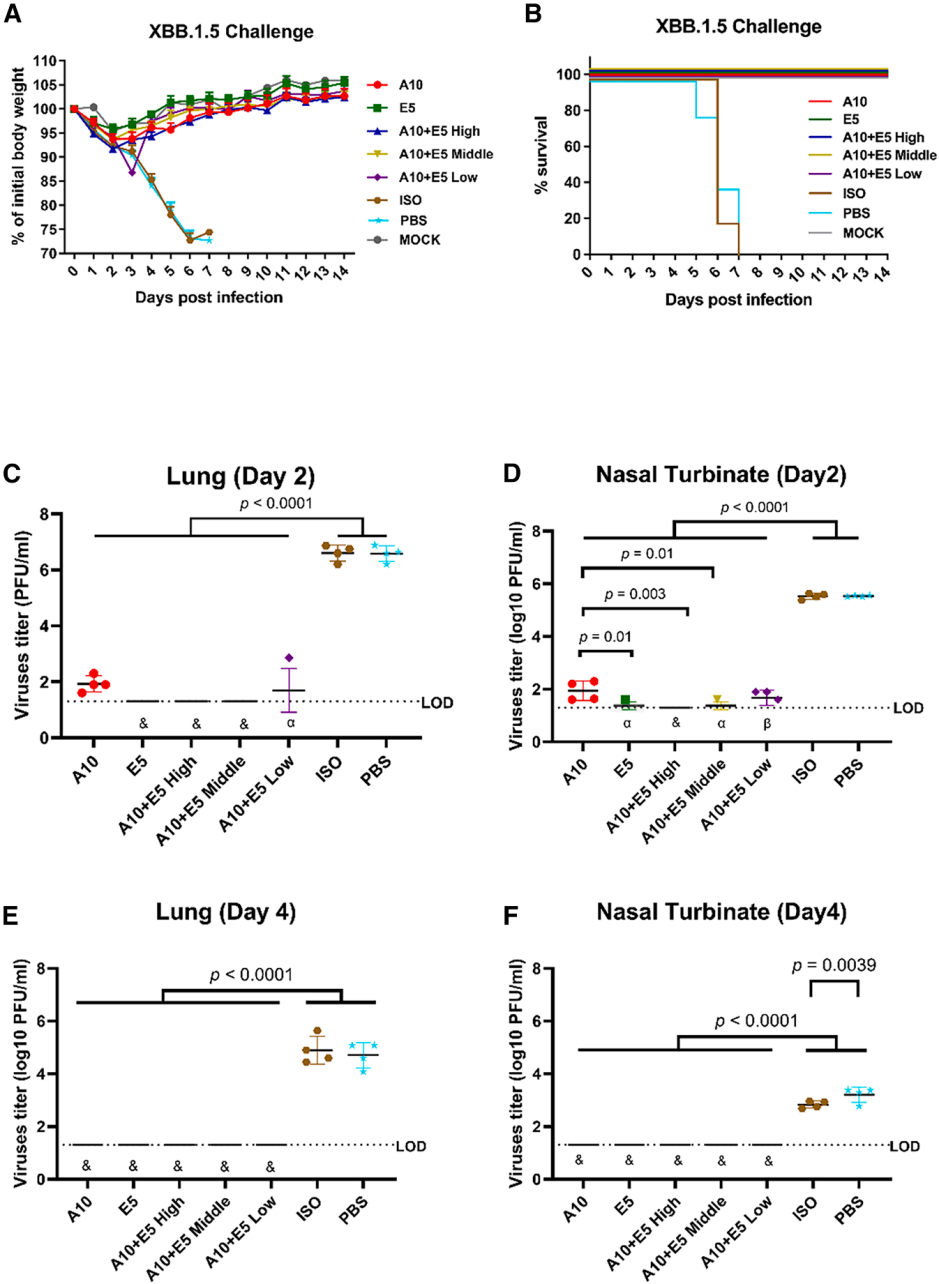
Prophylactic activity of hmAb against SARS-CoV-2 XBB1.5 K18 hACE2 transgenic mice were treated i.p. with either 20 mg/kg of 1324A10 (A10) or 1332E5 (E5), 20 mg/kg of 1324A10 plus 20 mg/kg 1332E5 (high), 10 mg/kg of 1324A10 plus 10 mg/kg 1332E5 (middle), 5 mg/kg of 1324A10 plus 5 mg/kg 1332E5 (low), 40 mg/kg isotype control IgG1 antibody (ISO, Bio X Cell, Lebanon, NH) or PBS. Mice were challenged 6 h later with 10^5^ PFU SARS-CoV-2 XBB.1.5. Body weight (A) and survival (B) were evaluated at the indicated days post-infection (*n* = 5 mice/group). Mice that lost >25% of their initial body weight were humanely euthanized. Error bars represent standard error of the mean (SEM) for each group of mice. Viral titers in the lungs (C and E) and nasal turbinates (D and F) at 2 and 4 days p.i. were determined by plaque assay in Vero AT cells (*n* = 4 mice/group/day). Symbols represent individual mice, bars indicate the mean of virus titers. Dotted lines indicate limit of detection. & indicates virus not detected in any mice from that group. α indicates virus detected in only one mouse from that group. β indicates only one mouse from that group had undetectable virus. Viral titers were analyzed by one-way ANOVA with Tukey’s multiple comparisons (*p* values are shown). Legend in Figure 3A is also for Figures 3C–3F.

Based on the *in vitro* neutralization and binding studies, 1324A10 and 1332E5 were chosen for *in vivo* assessment in a SARS-CoV-2 Omicron XBB1.5 lethal challenge model. K18 hACE2 transgenic mice were administered 1324A10, 1332E5, or a combination of the two, 6 h prior to challenge with XBB1.5 virus. Mice that received either PBS or isotype control antibody continued to lose weight (Figure 3A) until their humane endpoint on days 5–7 post-infection (Figure 3B), while mice treated with 1324A10, 1332E5 or their combination rebounded and continued to gain weight and survived until the termination of the experiment (day 14 post-infection).

Viral titers in the lungs of mice treated with 1324A10, 1332E5, or their combination were significantly lower than those in PBS- or isotype-control-treated mice, 2 days post-infection (Figure 3C). Mice treated with 20 mg/kg of 1332E5 or the combination of either 20 mg/kg or 10 mg/kg of 1324A10 and 1332E5 had no detectable virus in lungs 2 days post-infection, while only one mouse had detectable virus in the group administered 5 mg/kg of 1324A10 and 1332E5. Similarly, viral titers in nasal turbinate on day 2 post-infection were much lower in mice treated with 1324A10 and 1332E5 compared to PBS- and isotype-control-treated mice (Figure 3D). All mice treated with the high dose (20 mg/kg) of 1324A10 and 1332E5 had undetectable virus in the nasal turbinate 2 days post-infection, while all other treatments had at least one mouse with detectable virus. By 4 days post-infection, only PBS- and isotype control antibody-treated mice had detectable virus in the lungs (Figure 3E) and nasal turbinate (Figure 3F). These results indicate 1324A10 and 1332E5 alone and in combination are highly effective at preventing SARS-CoV-2 replication and lethality *in vivo*.

### Structural characterization of Fabs in complex with S^KP3.1.1^

**Figure 4:**
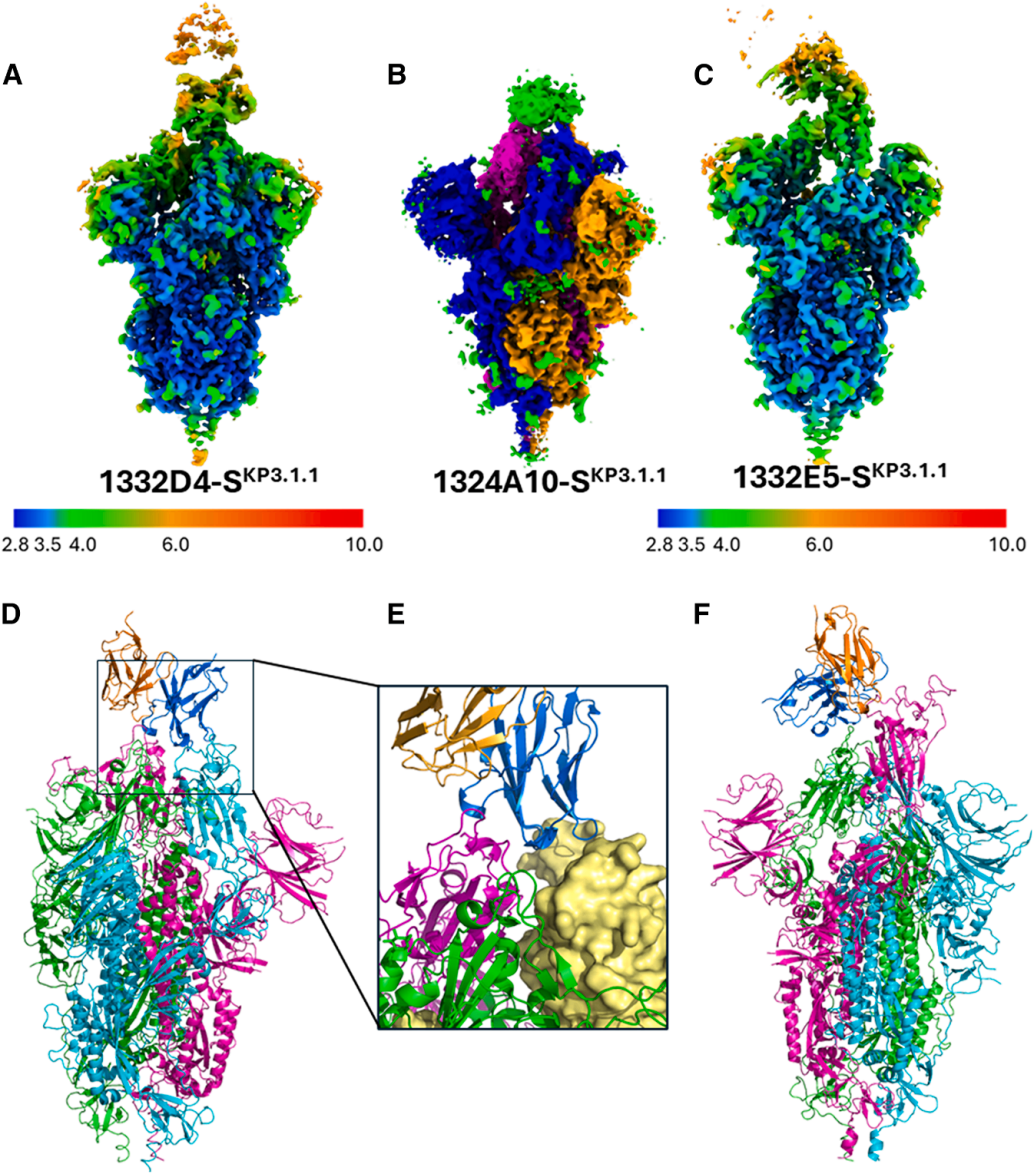
Density maps and overall structures of mAb-S^KP3.1.1^ complexes CryoEM density maps of S^KP3.1.1^ bound to 1332D4 (A), 1324A10 (B), and 1332E5 (C). 1332D4-S and 1332E5-S maps were refined to 3.26 Å and 3.23 Å resolution, respectively. Maps are colored by local resolution as shown in the bar at the bottom of the figure. The 1324A10-S^KP3.1.1^ map (B) was refined to 3.47 Å. S trimer without antibody or glycans was docked into the S^KP3.1.1^-1324A10 density that was colored green. The chains of the docked trimer model were colored orange, blue, and magenta. Density within 4 Å of the trimer model was colored by chain color using ChimeraX. Un-modeled density remains green and highlights glycans and putative Fab density near the accessible 440–446 loop on top (as shown in this figure) of S. Ribbon diagram of the S^KP3.1.1^-1332D4 complex (D). Heavy and light chains of the Fab are colored dark blue and orange, respectively. (E) 1332D4 binding to its primary RBD (magenta chain) prevents the adjacent RBD (RBD2), represented as a yellow surface, from adopting an ACE2 binding-competent conformation. (F) Ribbon diagram of the 1332E5-S^KP3.1.1^ complex, with the same coloring as in Figure D.

To further understand the varying neutralization profiles and *in vivo* activity, mAbs 1316C10, 1324A10, 1332D4 and 1332E5 were converted to Fab and incubated with recombinant S^KP3.1.1^. The resulting complexes were subjected to structure elucidation using cryo-electron microscopy (cryo-EM) (Figures 4 and S4–S8 and Table S1). Data collected for 1332D4-S^KP3.1.1^ (GSFSC 3.26 Å, Figure 4A) and 1332E5-S^KP3.1.1^ (GSFSC 3.23 Å, Figure 4C) yielded high-quality structures. Despite quality maps of S^KP3.1.1^, only poor density, presumed to be 1324A10 Fab, was observed near the 3-fold axis of S^KP3.1.1^ near the 1332D4 binding site (Figure 4B). There was no evidence of 1316C10 binding to S^KP3.1.1^, although a distorted S^KP3.1.1^ trimer was identified that may be related to how 1316C10 binds to S (Figure S8). The inability to visualize high-resolution structures of 1324A10 or 1316C10 Fabs was not due to poor Fab preparations, as neutralization assays confirmed that all Fabs used for cryo-EM retained binding/neutralization activity against SARS-CoV-2 PsV (Figure S9). To identify probable binding epitopes for 1324A10 and 1316C10, an SPR-based competition assay (Figure 2F) was performed using mAbs with known RBD binding epitopes (VIR-7229,^15^ class 1; RGN10933,^21^ class 1; RGN10987^21^ (1332D4 substitute), class 3: SA-55^22^ (1332E5 substitute), class 1/4; SC27,^13^ class 4/1 (see description later in discussion); CR3022,^23^ class 4; C68.C1,^16^ class 5). Both 1324A10 and 1316C10 blocked SA-55 (class 1/4), CR3022 (class-4), and SC27 (class 4/1) binding. This suggests 1324A10 and 1316C10 are broadly neutralizing mAbs that bind to RBD with class 4/1-like epitopes. The combined epitope mapping studies (Figures 2F and S1A) suggest 10 of 12 mAbs evaluated (83%) bind to the RBD through epitopes that overlap with class-4 and class-1 mAbs.

### Structure analysis of the 1332D4/S^KP3.1.1^ complex

**Figure 5:**
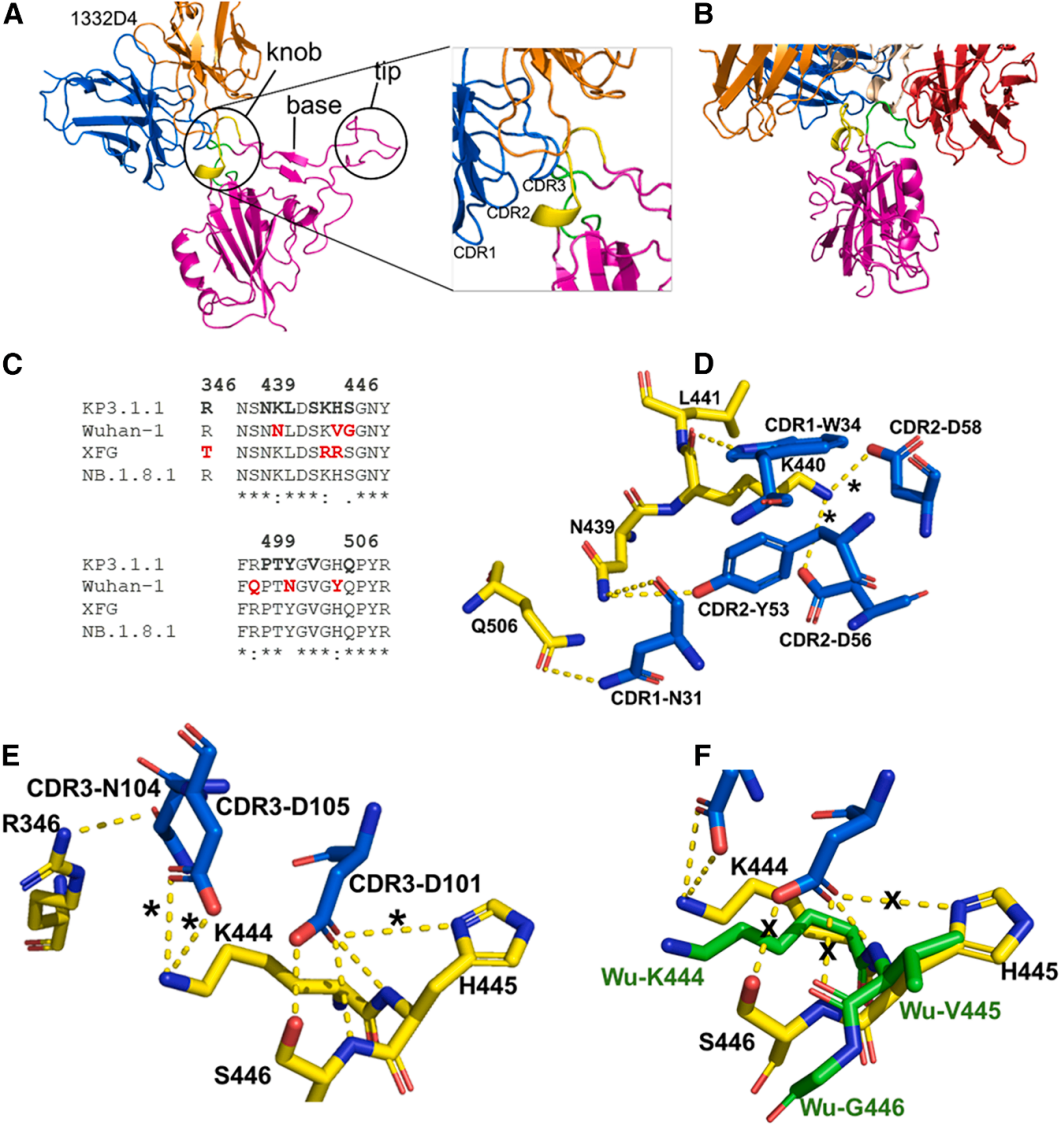
Molecular details of the RBD^KP3.1.1^-1332D4 interaction (A) Structure of the 1332D4-RBD^KP3.1.1^ complex highlighting key RBD (magenta) structural elements (knob, base, tip) with heavy and light chains colored blue and orange, respectively. The knob consists of RBD residues 439–447 (yellow) that form the main 1332D4 binding site, while knob residues 498–506 (green) form the main binding site of 1332E5. 1332E5 is colored red in (B), which highlights how the two epitopes are adjacent to one another. (C) Sequence differences within the 1332D4 epitope among recent Omicron variants. Residues that bury surface area in the complex are bolded. Red residues highlight residue differences relative to KP3.1.1. (D and E) Hydrogen bonds made between RBD^KP3.1.1^ (yellow) and 1332D4 residues (blue). Asterisks denote salt bridges in the interface. (F) Comparison of the KHS triplet in RBD^KP3.1.1^ (yellow) and the KVG triplet (green) from RBD^Wuhan-1^ (7U2D) with “x” denoting putative salt bridges disrupted in the modeled Wuhan-1/1332D4 complex.

**Figure 6:**
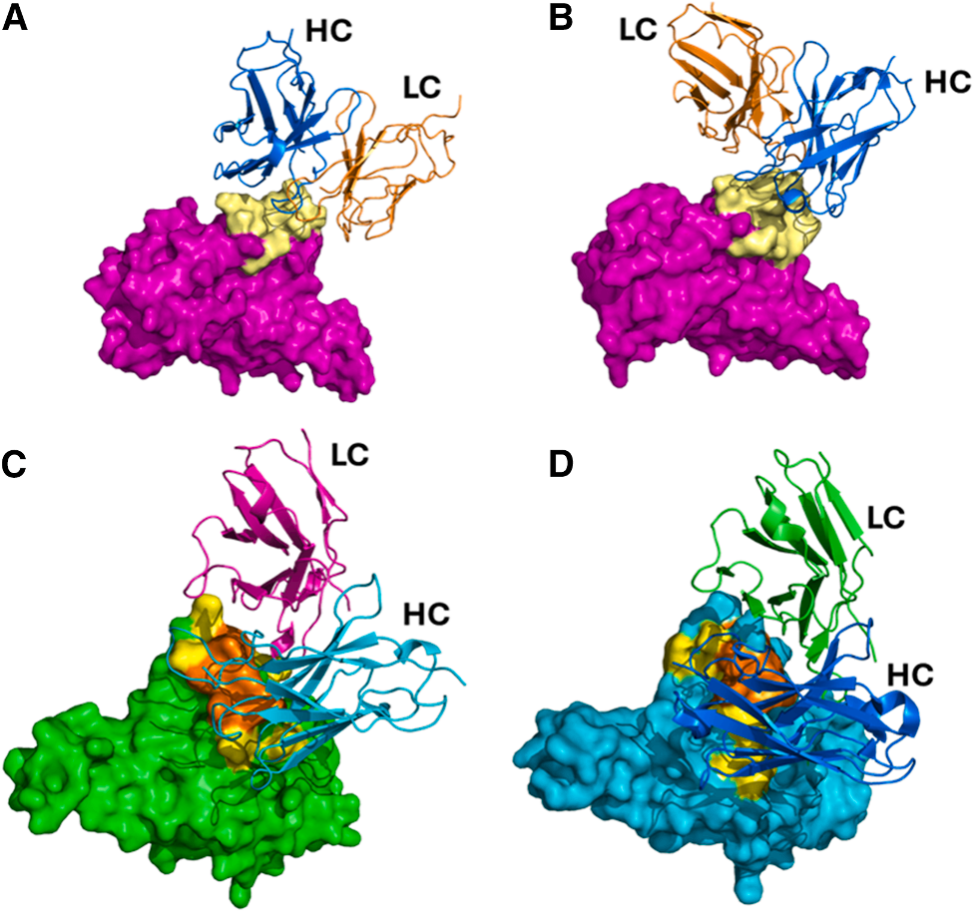
Similarity of isolated mAb epitopes to other antibodies RBD^KP3.1.1^-1332D4 complex (A) is compared to the RBD^Wuhan-1^-REGN10987 complex (6XDG) (B). RBD^KP3.1.1^-1332E5 complex (C) is highly similar to the RBD^Wuhan-1^-ADG20 complex (D). RBDs are represented as surfaces with the respective epitopes of the mAbs in yellow. For 1332E5 (C) and ADG20 (D), the epitopes are yellow, and knob residues 498–506 in the center of the epitopes are colored orange.

1332D4 binds to the RBD knob region (Figure 5A) on S^KP3.1.1^, exclusively in the closed conformation (Figures 4A and S5). 1332D4-S^KP3.1.1^ complex particles accounted for 56% of the total particles, while S^KP3.1.1^-only particles in the 1up (32%) and down (12%) conformations accounted for the remaining particles. To obtain greater detail of the 1332D4-S^KP3.1.1^ interface, local refinement of the RBD-1332D4 complex was performed, yielding a 3.56 Å resolution map (Figure S5). 1332D4 binds KP3.1.1 residues 346, 439–446, 499–503, and 506 almost exclusively (87% of bsa) with its heavy chain (Figures 5 and S10). 1332D4 buries 637 Å^2^ of surface area and forms 14 hydrogen bonds/salt bridges with RBD^KP3.1.1^. The most extensive 1332D4 interactions occur with Lys440 (Figure 5D) and the residue triplet Lys444-His445-Ser446 (Figure 5E) that interact with CDRH2 and CDRH3, respectively. Modeling studies suggest that changing the residue triplet KHS in KP3.1.1 to KVG, observed in the Wuhan-1 strain (Figure 5F), disrupts multiple hydrogen bonds and induces steric clashes with CDRH3 D101 due to the flip of the V445^Wuhan-1^ carbonyl oxygen (Figure 5F). Likewise, K440N would disrupt two salt bridges with CDRH2 D56 and D58 on the opposite end of the peptide. These observations are consistent with 1332D4’s inability to bind S^Wuhan-1^ or neutralize Wuhan-1 virus (Figures 1 and 2). The H445R mutation, recently identified in the XFG variant, would also induce significant steric clashes with 1332D4. H445R is the only disruptive XFG mutation in the 1332D4 epitope, which is consistent with the retention of some 1332D4 activity against XFG, compared to complete lack of binding and neutralization of Wuhan-1 (Figure 1D). The 1332D4 epitope is remarkably similar to REGN10987 (imdevimab, Figures 6 and S10). In particular, both mAbs lose neutralizing activity upon changes in amino acid position 445, H445R for 1332D4 and G445S for REGN10987.^21^

Potential 1332D4-S^KP3.1.1^ trimer interactions were also studied to understand the specificity of 1332D4 for the down conformation of S^KP3.1.1^. When bound to its primary epitope, the 1332D4 heavy chain is positioned over the adjacent RBD (RBD2), which sterically prevents RBD2 from adopting an ACE2 binding-competent 1up conformation (Figure 4E). The 1332D4-S^KP3.1.1^ model suggests 1332D4 makes minor contacts (e.g., buries less than 10 Å^2^ of surface area) with RBD2 residues Tyr-449, Lys-484, Pro-486, and Tyr-489. Overall, RBD2 and 1332D4 have complimentary contact surfaces that could form hydrogen bonds (currently ∼5 Å apart) depending on the conformation of RBD2 in the S trimer. The structure is consistent with the relatively weak binding (*K*D = 16.34 nM) between 1332D4 and the KP3 RBD (Figures 2D and 2E), whereas sensorgrams for the 1332D4-S^KP3.1.1^ interaction (Figure 2B) and the high potency of the mAb toward KP3.1.1 (Figure 1D) suggests a much higher affinity of the mAb for S^KP3.1.1^. Overall, the data suggest that 1332D4-RBD2 interactions are important, 1332D4 exhibits preferential selectivity for the down conformation of S^KP3.1.1^, and 1332D4 neutralizes virus by blocking the 1up conformation of S by sterically occluding S from reaching ACE2 on the cellular surface. In addition, the 1332D4 epitope overlaps the ACE2 binding site that might provide an additional mechanism of neutralization for 1324D4 (Figure S11).

### Structure analysis of the 1332E5/S^KP3.1.1^ complex

**Figure 7:**
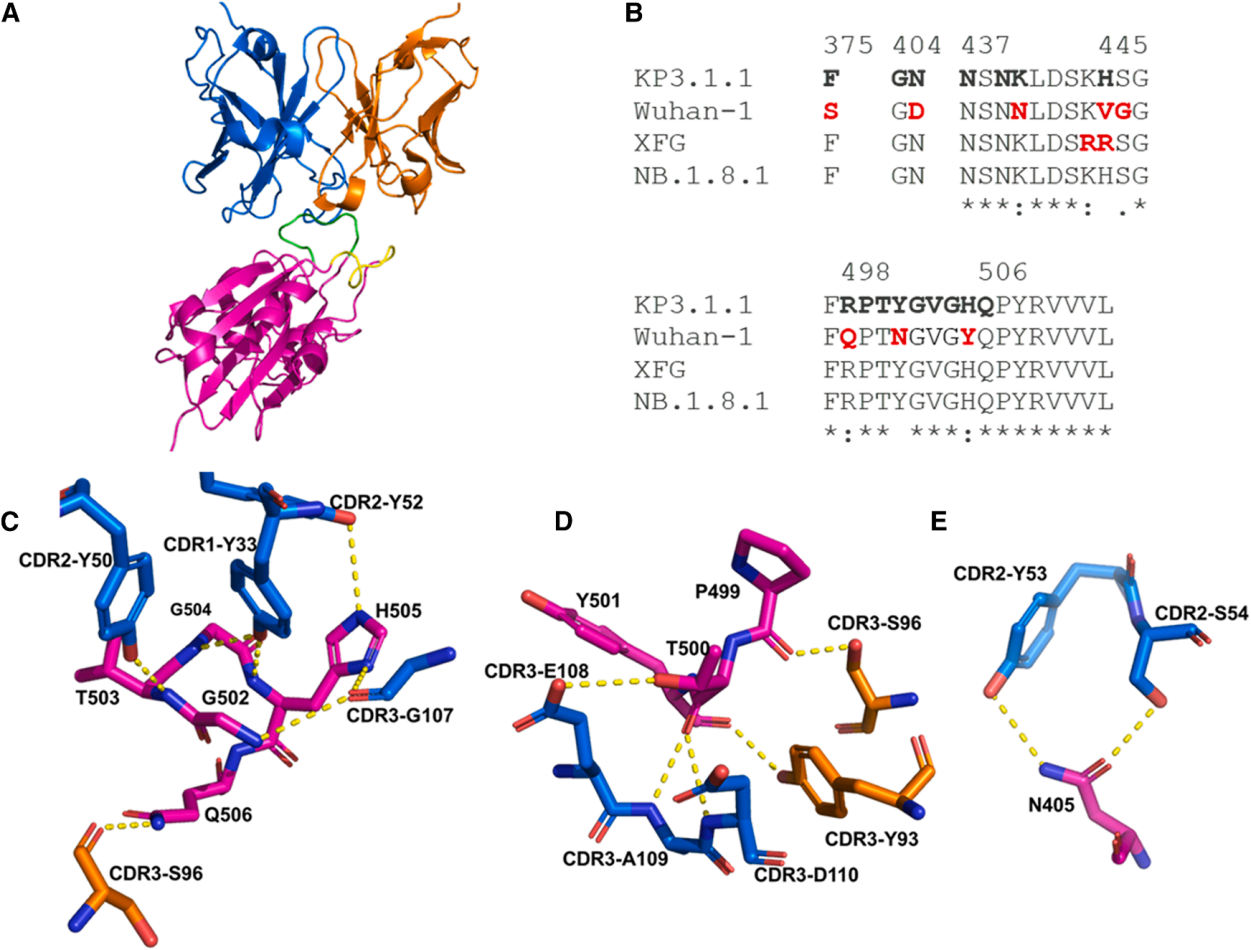
Molecular details of the 1332E5-RBD^KP3.1.1^ interaction (A) Structure of the 1332E5-RBD^KP3.1.1^ with RBD (magenta) with knob residues 498–506 and 439–447 colored green and yellow, respectively. (B) Sequence of the 1332E5 epitope (bold residues) with residue differences of variants discussed in the text in red. (C–E) Molecular contacts in the 1332E5-RBD^KP3.1.1^ interface. Hydrogen bonds are shown as yellow dashes. For all figures, heavy and light chains of 1332E5 are colored blue and orange, respectively.

1332E5 binds to S^KP3.1.1^ in the 1up conformation (Figures 4C and 4F). In contrast to 1332D4 data, all particles in the 1332E5-S^KP3.1.1^ dataset were bound by Fab, albeit with varying density quality (Figure S6). 1332E5 epitope overlaps the ACE2 binding site, suggesting direct occlusion of ACE2 binding is its major mechanism of mAb-mediated viral neutralization (Figure S11). Local refinement of 1332E5-RBDKP3.1.1 was performed to identify the details of the interface (Figures 7 and S6). 1332E5 binds to the knob region of the KP3 RBD, essentially opposite 1332D4 (Figure 5B). A total of 16 KP3.1.1 residues (726 Å^2^) on three main RBD residue segments (404–405, 439–445, and 498–506) form the 1332E5 binding site (Figure 7 and Table S2). KP3.1.1 residues 499–506 make the most extensive interactions with 1332E5, especially Thr-50, Val-503, His-505 that bury 114 Å^2^, 88 Å^2^, and 82 Å^2^ of surface area, respectively. 1332E5 CDRs are enriched in tyrosines (CDRH1,Y33; CDRH2 Y50, Y52; and CDRL3, Y93) that along with CDRH3 residues G107, E108, A109, D110 form multiple hydrogen bonds with the peptide (Figures 7C–7E). Sequence and structural comparisons identified eight amino acid differences between KP3.1.1 and Wuhan-1 RBDs (Figure 7B). Modeling suggests the most disruptive mutation in Wuhan-1 S would be Y505 (H505 in KP3.1.1) that causes steric clashes between 1332E5 heavy chain residues W105 and G107. In contrast to 1332D4, the 1332E5 epitope is not sensitive to mutations in the 444–445 region and retains significant activity against recent viral variants NB.1.8.1 and XFG (Figure 1D).

### 1332E5 is an RBD knob^498-506^-targeting mAb

**Figure 8:**
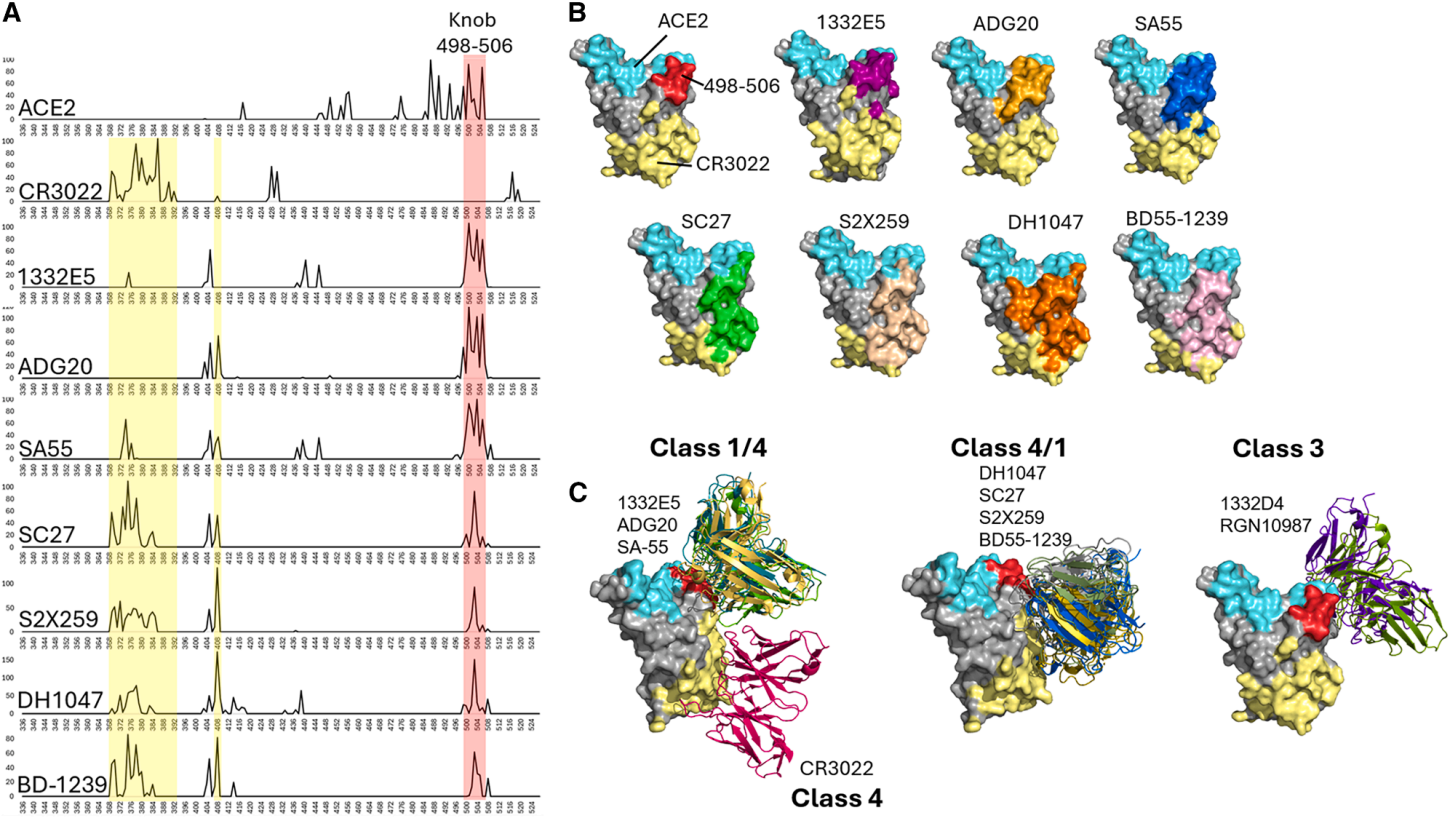
Two distinct 1/4 antibody classes (A) Buried surface area (y axis) traces vs. RBD residue (x axis) for ACE2 (6M0J) and mAbs CR3022 (6W41), 1332E5, ADG20 (7U2D), SA55 (7Y0W), SC27 (8VIF), S2X259 (7RAL), DH1047 (7SG4), and BD55-1239 (7WRL). Knob^498-506^ (red) and CR3022 (yellow) regions are highlighted. (B) RBD contact surfaces highlighting ACE2 binding site (cyan), knob^498-506^ (red), CR3022 epitope (yellow). MAb epitopes distinguish those that predominantly target knob^498-506^ (1332E5, ADG20, SA55) versus those that predominantly contact the CR3022 epitope (SC27, S2X259, DH1047, and BD55-1239). (C) Superposition of MAbs based on their distinct classifications. For comparison, class 3 mAbs 1332D4 and RGN10987 (6XDG) that target knob^439-446^ are shown, and the class-4 mAb, CR3022.

The central epitope of mAb 1332E5 is comprised of RBD knob residues 498–506 (Figure 8). This epitope has been called the F3 site^22^ and the RBS-D site.^24^ 1332E5 binding requires S to adopt an up conformation where it predominantly targets residues within the ACE2 binding site. However, three 1332E5 contact residues adjacent to knob^498-506^ (F374, T376, and R408) are shared with the CR3022 binding epitope, which is the prototypical class-4 mAb.^25^ For this reason, 1332E5 is generically described as a class 1/4 antibody. Other mAbs with very similar epitopes to 1332E5 include ADG20^24^ and its derivatives, including the clinically relevant VYD222 (pemivibart),^17^ as well as SA55.^22^ All three mAbs exhibit high potency viral inhibition due to extensive overlap with the ACE2 binding site (Figure S11), but exhibit varying degrees of binding breadth to SARS-CoV and SARS-CoV-2 variants (Figures 2 and S1). ADG20 binds and neutralizes SARS-CoV and SARS-CoV-2 Wuhan-1, but loses activity rapidly against Omicron variants. 1332E5 exhibits neutralization breadth against Omicron variants, but has no activity against SARS-CoV or Wuhan-1. Finally, SA55 has maintained broad binding and potent neutralization of SARS-CoV through the latest Omicron variants.

1332E5 and other class 1/4 knob^498-506^-targeting mAbs are distinguished from a related group of mAbs that primarily target the class 4 epitope, with limited contacts (mainly residues 502–504) within knob^498-506^ (Figure 8). mAbs targeting this epitope include S2X259,^26^ DH1047,^27^ BD55-1239,^22^ and SC27.^13^ Epitope mapping (Figure 2F) suggests 1316C10 and 1324A10 also fall into this category of mAbs. These mAbs are also referred to as class 1/4 mAbs, yet as shown in Figure 8, they target a very distinct epitope, suggesting further clarity in the current nomenclature would be helpful. Simply, we suggest these mAbs should be classified as 4/1 mAbs to distinguish their distinct class-4-biased epitope.

### Sequence conservation in the SARS-CoV-2 RBD knob^498-506^

**Figure 9:**
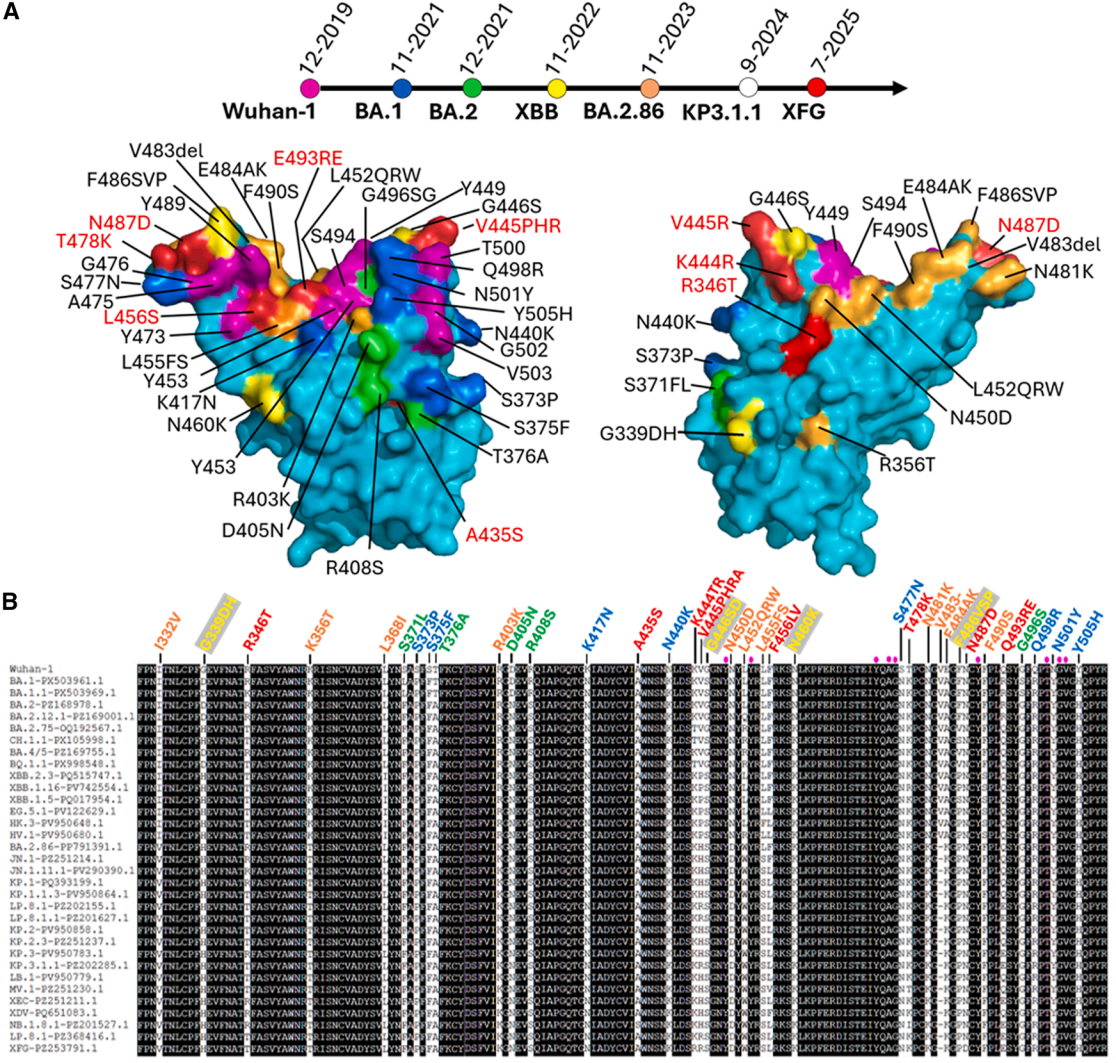
RBD amino acid sequence conservation among major SARS-CoV-2 Omicron variants (A) Approximate timeline (through XFG) for the emergence of major SARS-CoV-2 Omicron variants. The colors on the timeline correspond to mutations on the Wuhan-1 RBD (6M0J) surface (cyan), with the ACE2 binding site colored magenta. The figure highlights the conserved nature of the knob^498-506^. (B) RBD sequence alignment of major variants with mutations highlighted. Magenta dots correspond to ACE2 binding residues that have not undergone mutation.

The evolutionary trajectory of SARS-CoV-2 S is to optimize viral fitness by modulating competing needs of the virus to evade the immune system while maintaining and/or increasing infectivity and transmission. RBD mutations in variant viruses have played a large role in both antibody immune evasion and infectivity while preserving ACE2 binding. To study this evolution, mutations in major emerging Omicron variants from BA.1 through XFG were mapped chronologically onto the RBD structure (Figure 9). The analysis shows all but three residues in the RBD knob^498-506^ retain their original Wuhan-1 amino acid sequence, and the three mutated residues (Q498R, N501Y, and Y505H) have been conserved since the original BA.1 variant. These findings provide insight into the diverse specificities of knob^498-506^-targeting mAbs. 1332E5 and ADG20 both target N501Y and Y505H mutations that were changed in the original Omicron variant. This explains why 1332E5 cannot recognize pre-Omicron variants and why ADG20 does not recognize post-Omicron variants. In contrast, SA55 has more limited contacts with 501 and 505 and forms more extensive contacts around V503, which is conserved in the original Wuhan-1 virus. Although not found in nature, deep mutational scanning confirms RBD mutations at 503 and 504 disrupt SA55 binding.^28^

## Discussion

The goal of vaccination against SARS-CoV-2 is to induce broadly neutralizing antibodies (bNAbs) that protect against current and future viral variants. Many bNAbs, such as those that target the spike S2 domain, exhibit substantial breadth but low potency.^29^^,^^30^ As a result, tremendous effort has been made to induce and characterize potent RBD-targeting bNAbs generated by natural infection and/or through vaccination. The isolation and development of bNAbs holds great clinical potential for prophylactic and/or therapeutic protection against the virus in targeted populations including immunocompromised individuals. Unfortunately, multiple RBD-targeting neutralizing Abs exhibiting high potency have been developed for clinical use only to be rendered ineffective by the continued evolution of the virus.^31^

Pressure from the natural immune response, vaccination, and even mAb therapies can drive mutations in SARS-CoV-2 RBD.^32^ 1332D4, isolated in this study, provides an example of the type of mAbs that can drive viral escape processes. 1332D4 is a class-3 mAb that makes extensive contacts with the exposed RBD knob epitope, residues 439–446. During the sample collection period, the epitope targeted by 1332D4 underwent mutational diversity, with H445^KP3.1.1^ replaced by R445 in the XFG variant virus. While 1332D4 exhibits single-digit potency for KP3.1.1, the mAb has no neutralizing activity against XFG, harboring the H445R mutation.

To limit mutational viral escape mechanisms, we and others have argued that RBD mAbs should target conserved amino acids and functionally important regions of the virus that, if altered, disrupt virus viability.^28^^,^^33^ 1332E5 targets the highly conserved RBD knob^498-506^ that forms part of the ACE2 binding site and shares this epitope with ADI5668, ADG20 and derivatives (e.g., VYD222), as well as SA55. These mAbs can be described as class 1/4 knob^498-506^-targeting mAbs and distinguished from a related group of mAbs that predominantly target the class-4 epitope and make limited contacts (mainly residues 502–504) with knob^498-506^. mAbs targeting this epitope include S2X259,^26^ DH1047,^27^ BD55-1239,^22^ and SC27.^13^ Although these mAbs are referred to as class 1/4 mAbs, they target a very distinct class-4-biased epitope that can be highlighted by defining them as class 4/1 mAbs.

As pointed out in our analyses, class 1/4 knob^498-506^-targeting mAbs exhibit variable breadth in binding and neutralization of viral variants biased toward pre-Omicron (ADG20), post-Omicron (1332E5), or broad neutralization of pre- and post-Omicron variants (SA55). These differences are determined by specific mAb interactions with knob^498-506^. 1332E5 and ADG20 are sensitive to mutations at RBD positions N501Y and Y506H that changed with Omicron, while SA55 binding is disrupted by mutations in knob residues 502–504, that are highly conserved in SARS-CoV and SARS-CoV-2 variants. Notably, SA55 and many class 4/1 mAbs are sensitive to mutations in the same 502–504 region of the RBD knob. The added benefit of class 1/4 mAb SA55 over class 4/1 mAbs is the greater overlap with the ACE2 binding site and increased accessibility of the epitope, which both can increase mAb potency. Overall, class 1/4 and 4/1 mAbs that target the knob^498-506^ region of RBD can exhibit broad and potent neutralizing activity against multiple sarbecoviruses and may be optimally suited for clinical use in immunocompromised individuals.

The unique broad specificity of knob-targeting mAbs suggests it is important to understand how this class of broadly neutralizing Abs is induced. ADI55668, the initial scaffold for the development of ADG20/VYD222, was isolated from a SARS-CoV-infected individual that had prior exposures to endemic human coronaviruses (HCoVs).^34^ SA55 was also isolated from a SARS-CoV-2-recovered individual that was subsequently vaccinated with ancestral SARS-CoV-2.^22^ In contrast, 1332E5 was isolated from an individual, presumably infected with the KP3.1.1 variant who had been previously vaccinated. Similarly, SARS-CoV-2-infected individuals subsequently vaccinated gave rise to class 4/1 mAbs,^13^ further emphasizing the importance of multiple antigen exposures to elicit bNAbs. Future studies need to develop appropriate antigens to optimize the generation of bNAbs despite diverse infection/vaccination histories.

The discovery of 1332E5 further suggests that knob^498-506^-targeting mAbs may be more prevalent now (2024 onward) than early in the COVID-19 pandemic. This is potentially important if immune pressure from Abs targeting the RBD-knob region results in knob escape mutations. Notably, the RBD knob region has been “stable” since significant mutational changes occurred with the emergence of Omicron in late 2021 (e.g., N501Y, Y505H). In the case of 1332E5, reversion to the ancestral Wuhan-1 knob epitope would entirely disrupt its activity. However, the likelihood of reemergence of older variants may be limited due to the high transmissibility of current dominant lineages. However, we note the increase in prevalence of BA.3.2, which reverts H505 to Y505 and would disrupt 1332E5-like mAbs.^35^^,^^36^ Overall, this study has identified a highly potent mAb with broad specificity for Omicron variants and characterized the conserved RBD knob^498-506^ epitope for broad binding and neutralization of diverse sarbecoviruses, including potential future emerging variants.

### Limitations of the study

This study uses a small number of individuals with incomplete infection/vaccination histories. Thus, the prevalence of knob-targeting Abs or their specific infection/vaccination history that led to the induction of 1332E5 is unknown. The cryo-EM reconstructions for the full-length S/Fab complexes exhibit density in RBD and NTD that do not provide unambiguous sidechain positions of some residues. This is due to inherent flexibility in S1 of the KP3.1.1 S. Despite higher reported resolution, other structures of KP3.1.1 exhibit similar density in the S1 domain.^37^ Local refinements recover high-quality maps for RBD-Fab complex interactions, but the details of inter-protomer (e.g., RBD NTD) amino acid residues and their contacts in the S1 trimer cannot be defined unambiguously in the experimental maps. This study characterized the structures of Fab-S complexes and did not evaluate structures of S bound to intact antibody. This limits our ability to understand some functions of the antibodies such as bi- or multi-valent binding and steric occlusion mechanisms.

## Resource availability

### Lead contact

Further information and requests for resources and data should be directed to and will be fulfilled by the lead contact, Mark R. Walter (walter@uab.edu).

### Materials availability

Antibodies identified in this report will be shared with a completed materials transfer agreement.

### Data and code availability

• Coordinates for the 1332D4 Fab in complex with the Omicron KP3.1.1 spike have been deposited to the Protein DataBank (PDB) as PDBID 11HO (Fab-trimeric S) and PDBID: 11HM (locally refined Fab-RBD). Coordinates for the 1332E5 Fab in complex with the Omicron KP3.1.1 spike have been deposited to the Protein DataBank (PDB) as PDBID 11IS (Fab-trimeric S) and PDBID: 11IT (locally refined Fab-RBD). Cryo-EM maps have been deposited to the Electron Microscopy DataBank under accession codes EMD-75698, EMD-75696, and EMD-75725, and EMB-75726.
• All data reported in this paper will be shared by the lead contact upon request.
• This paper does not report any original code.
• Any additional information required to reanalyze the data reported in this paper is available from the lead contact upon request.

## Acknowledgments

We are grateful to the clinical research staff who enabled this project and to the assistance of the UAB Flow Cytometry and Single Cell Core Facility, UAB Multidisciplinary Molecular Interaction Core Facility, and UAB cryo-EM Facility. We thank BEI Resources for providing reagents. We thank Dr. Clint Florence, Program Officer, Office of Biodefense, Research Resources, and Translational Research at NIH/NIAID/DMID/OBRRTR/RRS for providing SARS-CoV-2 Omicron XBB1.5 and JN.1 variants. We are most grateful for the participation of the study volunteers. Funding for this work was provided by institutional support from UAB (to J.J.K.) and Texas Biomedical Research Institute (to L.M.-S.), and the National Institutes of Health (grant no. 1R01AI161175 to J.J.K., L.-M.S, and M.R.W.). The UAB cryo-EM Facility (RRID: SCR_025450) is supported by the UAB Institutional Research Core Program and O’Neal Comprehensive Cancer Center (NIH grant no. P30CA013148), with additional funding from NIH grant no. S10OD024978. Funders had no role in study design, data collection and analysis, decision to publish, or preparation of the manuscript.

## Author contributions

J.J.K., L.M.-S., and M.R.W. conceived the study. M.S.P., A.R.B., A.M.B., and J.J.K. isolated and screened the mAbs. M.S.P., S.P., and M.R.W. produced the recombinant proteins and conducted. S.P.R., J.L.K., and M.R.W. conducted the cryo-EM analysis. Y.M., S.H.M., and L.M.-S. conducted the *in vitro* and *in vivo* live virus assessments. G.C.I. provided reagents and expertise. P.A.G. and N.B.E. acquired clinical specimens. M.S.P. conducted the pseudovirus neutralization assays. J.J.K., L.M.-S., and M.R.W. supervised the work. M.S.P., J.J.K., L.M.-S., and M.R.W. wrote the manuscript. All authors have read and approved the manuscript.

## Declaration of interests

M.S.P., Y.M., S.P., A.R.B., N.B.E., P.A.G., L.M.-S., J.J.K., and M.R.W. are co-inventors on a patent application that includes claims related to the mAbs described in this manuscript.

## STAR★Methods

### Key resources table

**Table:** 

REAGENT or RESOURCE	SOURCE	IDENTIFIER
**Antibodies**		

Brilliant Violet 510™ anti-human CD3 Antibody (OKT3)	Biolegend	Cat#: 317331; RRID: AB_2561376
Brilliant Violet 510™ anti-human CD4 Antibody (OKT4)	Biolegend	Cat#: 317444; RRID: AB_2561866
Brilliant Violet 510™ anti-human CD14 Antibody (S3D3)	Biolegend	Cat#: 367124; RRID: AB_2716229
PE/Cyanine7 anti-human CD27 Antibody (O323)	Biolegend	Cat#: 302837; RRID: AB_2561918
PE/Cyanine7 anti-human IgD Antibody (IA6-2)	Biolegend	Cat#: 348209; RRID: AB_10683460
Peroxidase-conjugated AffiniPure Goat anti-Human IgG Fcγ Fragment	Jackson ImmunoResearch	109-035-008; RRID: AB_2337579
1301B7	Piepenbrink et al.^14^	N/A
C68.61	Guenthoer et al.^38^	N/A
SA55	Cao et al.^22^	N/A
SC27	Voss et al.^13^	N/A
VYD222	Yuan et al.^17^	N/A
VIR7229	Rosen et al.^15^	N/A
InVivoPlus human IgG1 isotype control	Bio X Cell	Cat#: BP0297; RRID: AB_2894747
1C7C7	Drs. Thomas Moran and James Duty, Ichan School of Medicine, Mount Sinai.	1C7C7

**Bacterial and virus strains**		

DH10B chemically competent cells	Intact Genomics	Cat#: 1011-24
SARS-CoV-2 WA.1	Center for Disease Control and Prevention/BEI Resources	Cat#: NR-52281
SARS-CoV-2 XBB1.5	BEI Resources	NR-59104
SARS-CoV-2 KP.3	BEI Resources	NR-59892
Pseudotyped SARS-CoV-2 (Wuhan-1)	BEI Resources	NR-53816
Pseudotyped SARS-CoV-2 (JN.1)	This study	NA
Pseudotyped SARS-CoV-2 (NB1.8.1)	This study	NA
Pseudotyped SARS-CoV-2 (XFG)	This study	NA

**Biological samples**		

Human peripheral blood/PBMC	This study	NA

**Chemicals, peptides, and recombinant proteins**		

BirA biotinylation enzyme	Avidity	Bulk BirA
Nucleocapsid protein – PE	Sino Biologicals	40588-V27B-B
SureBlue	SeraCare Life Sciences, Inc.	51200077
Recombinant SARS-CoV-2 JN.1 spike protein	This study	N/A
Recombinant SARS-CoV-2 KP.3 spike protein	This study	N/A
Recombinant SARS-CoV-2 KP.3 RBD-2LL-ACE2 fusion protein	This study	N/A
Recombinant SARS-CoV-2 Wu-1 spike protein	BEI Resources	Cat#: NR-52307
Recombinant SARS-CoV spike protein	BEI Resources	Cat#: NR-55704
Recombinant SARS-CoV-2 Spike RBD (Wuhan-1)-Fc fusion	This study	N/A

**Critical commercial assays**		

qScript cDNA Synthesis Kit	QuantaBio	Cat# 95047-100
Hot Start *Taq* DNA Polymerase	New England Biolabs	Cat# M0495L
EconoTaq PLUS GREEN 2X Master Mix	LGC Genomics	Cat# 300331
iProof™ High-Fidelity DNA Polymerase	Bio-Rad	Cat# 1725302
Biacore Sensor Chip CM5	Cytiva Life Sciences	Cat#: BR100399
Biacore Sensort Chip NTA	Cytiva Life Science	Cat#: 28994951
Mouse antibody (Fc) capture kit	Cytiva Life Sciences	Cat#: BR100838
Human antibody (Fc) capture kit	Cytival Life Sciences	Cat#BR100839
Bright-Glo™ Luciferase Assay System	Promega	Cat#: E2620

**Deposited data**		

1332D4-S(KP3.1.1) Structure	This study	PDBID 11HO
1332D4-RBD(KP3.1.1) Structure	This study	PDBID 11HM
1332E5-S(KP3.1.1) Structure	This study	PDBID 11IS
1332E5-RBD(KP3.1.1) Structure	This study	PDBID 11IT

**Experimental models: Cell lines**		

HEK293T	ATCC	CRL-3216
African green monkey kidney epithelial cells (Vero E6)	ATCC	CRL-1586
Vero E6 expressing Human ACE2 and TMPRSS2 (Vero AT)	BEI Resources	NR-54970
HEK293T cells expressing human ACE2	BEI Resources	NR-52511

**Experimental models: Organisms/strains**		

B6.Cg-Tg(K18-ACE2)2Prlmn/J [K18-ACE2 mouse]	Jackson Laboratory	RRID:IMSR_JAX:034860

**Oligonucleotides**		

Primers for Expression Cassette Fragments	This study	See Table S3
Primers for second round PCR for variable heavy, kappa, and lambda fragment generation	This study	See Table S3

**Software and algorithms**		

Prism (V10)	Graphpad	https://www.graphpad.com/
EPU software	N/A	Thermofisher.com
cryoSPARC v4.5.3	cryoSPARC	https://cryosparc.com/
segger	Pintilie et al.^39^	https://www.cgl.ucsf.edu/chimerax/
ChimeraX	RBVI	https://www.cgl.ucsf.edu/chimerax/
Phenix	Phenix	https://www.phenix-online.org/
PyMol	PyMOL by Schrödinger	https://pymol.org: RRID: SCR_000305

### Experimental model and study participant details

Peripheral blood was collected at the University of Alabama at Birmingham (UAB) from adult convalescent individuals approximately 1–6 weeks following infection with SARS-CoV-2 from April 2023 to August of 2024.

All procedures and methods involving human samples were approved by the Institutional Review Board for Human Use at the University of Alabama at Birmingham (IRB-160125005). Written or oral informed consent was obtained from the participants. All experiments were performed in accordance with relevant guidelines and regulations.

### Method details

#### B cell isolation

Peripheral blood mononuclear cells (PBMC) were isolated by density gradient centrifugation and cryopreserved. Spike (S) proteins of JN.1 or KP.3.1.1 Omicron variants were generated in the TurboCHO system of GenScript (Piscataway, NJ). Both proteins contain C-terminal T4 fibritin trimerization domains, his8 tags and Avi biotinylation tags. Similar to that described by Piepenbrink et al.,^14^ a KP.3 RBD-2LL-ACE2 (RBD residues 327–511, 18-residue linker, ACE2 residues 20–787 with C-terminal his6 and avi tags.) fusion protein complex was expressed in the TurboCHO system of GenScript and purified by nickel affinity chromatography. The purity of proteins was confirmed by SDS-PAGE under reducing and non-reducing conditions by GenScript. The proteins were biotinylated in-house using biotin ligase (BirA, https://www.avidity.com/) and then streptavidin tetramers (4 mol protein to 1 mol streptavidin) formed for B cell isolation experiments. Cryopreserved cells were thawed and then stained for flow cytometry similar as previously described,^40^ using anti- CD19-APC-Cy7 (SJ25C1, BD Biosciences), SARS-RBD-BV421, JN.1 or KP.3.1.1 S (dual labeled - Alexa Fluor 488 and -AlexaFluor647), RBD-ACE2FP or KP.3- RBD-2LL-ACE2FP-BV786, CD3-BV510 (OKT3, Biolegend), CD4-BV510 (OKT4, Biolegend), CD14-BV510 (63D3, Biolegend), CD27-PE/Cy7 (O323, Biolegend) or IgD-PE/Cy7 (IA6-2, Biolegend), Annexin V-PerCP-Cy5.5 (Biolegend), SA-BV421 (Biolegend), SA-AlexaFluor647 (Biolegend), SA-BV786 (Biolegend), SA-PE (Biolegend) linked to SARS-CoV-2 Nucleocapsid protein (NC-PE, Sino Biologicals), and Live/Dead aqua (Thermo Scientific/Molecular Probes).

#### Monoclonal antibody production

Single B cells were sorted using a FACSMelody (BD Biosciences) into 96-well PCR plates containing 4 μL of lysis buffer (0.5× PBS with 10 mM DTT (Invitrogen), and 8 U RiboLock (ThermoFisher) RNase inhibitor) as previously described.^41^ Plates were immediately frozen at −80°C after sorting until thawed for reverse transcription using 16 μL of Q-Script (QuantaBio, Beverly, MA, #95047-100) reaction mix and thermocycler settings of 22°C for 5 min, 42°C for 30 min, and 85°C for 5 min before cooling to 4°C. The resulting cDNA product was used in nested PCR performed for IgH, Igλ, and Igκ variable gene transcripts using a modified process of Liao et al.^42^ which allowed for a more streamlined cloning approach and robust antibody expression. Briefly, 4 μL of cDNA product was utilized in the first round for each of the Heavy, Kappa, and Lambda (outer PCR) using Hot Start Taq DNA Polymerase and reaction buffer (New England Biolabs, #M0495L) in a 40 μL reaction using 40 cycles of the thermocycling conditions of Liao et al.^42^ The second round (inner) of PCR was modified to allow quick cloning into the respective Ig Heavy, Ig Kappa and Ig Lambda expression vectors originally described by Tiller et al.^43^ For this, new primers were generated (Table S3) and used at a final concentration of 0.05 μM each in separate heavy, kappa, and lambda reactions with 4 μL of first round PCR products in a 40 μL reaction mix (EconoTaq Plus Green, Lucigen). Forty cycles of the following thermocycling conditions were used after an initial denaturing step of 95°C for 5 min. Heavy: 95°C for 30 s, 52°C for 30 s, and 68°C for 60 s; Kappa: 95°C for 30 s, 45°C for 30 s, and 68°C for 60 s; Lambda: 95°C for 30 s, 50°C for 30 s, and 68°C for 60 s. A final extension was carried out at 68°C for 10 min before cooling to 10°C. The resulting PCR products were separated on a 1% agarose Tris-Borate-EDTA gel containing SYBR Safe DNA gel stain (Thermo Scientific) using GeneRuler 1 kb plus (Thermo Scientific) as a size marker. DNA bands around 500 bp were cut out and cleaned up using the E.Z.N.A Gel Extraction Kit (Omega Biotek #D2500; Norcross, GA).

Clean PCR products were used in expression cassette generation. Briefly, cassette fragments for CMV and constant regions for heavy, kappa, and lambda immunoglobulins were generated from their respective expression plasmids using target specific primers (See STAR Methods). Multiple 50 μL reactions were prepared for each fragment generation using iProof High Fidelity Polymerase (Bio-Rad, # 1725302), 0.4 μM of each primer pair, and 10 ng of the respective plasmid DNA. Thermocycling conditions were set for an initial denaturing step of 98°C for 30 s, 30 cycles of 98°C for 10 s, 60°C for 30 s, and 72°C for 1 min followed by a final extension of 10 min at 72°C and cooling to 12°C. Cassettes were generated by combining iProof high fidelity master mix with 10 ng CMV fragment, 10 ng corresponding constant fragment, Forward CMV frag primer and Reverse Cassette primer (both at 0.4 μM final concentration) and 2 μL gel extracted PCR product in a 50 μL reaction. Thermocycling conditions were run for an initial denaturing at 98°C for 60 s, 3 cycles of 98°C for 15 s, 62°C for 20 s, and 72°C for 60 s followed by 25 cycles of 98°C for 15 s, 58°C for 20 s, and 72°C for 60 s. A final extension was carried out at 72°C for 10 min followed by cooling to 12°C. PCR products were cleaned up using a MiniElute 96 UF filter (Cat # 28051, Qiagen).

For cassette expression, 28,000 HEK293T cells/well were grown at 37°C with 5% CO_2_ in 96-well, flat bottom plates, with DMEM supplemented with 10% Hyclone Fetal Clone II (Cytiva) and antibiotics and antimycotics (Corning) until 60–80% confluent when media was exchanged for DMEM with 2.5% Hyclone Fetal Clone II and antibiotics and antimycotics. Two μL of each corresponding heavy and light chain expression cassettes was mixed with 0.25 μL of jetPRIME® and 5 μL transfection buffer (Polyplus, Illkirch-Graffenstaden, France) and incubated for 10 to 20 min at room temperature before adding the mixture to wells containing HEK293T cells. Supernatants were harvested five days later and used in rapid screening against spike proteins by ELISA (described below). Those that had OD values greater than 7 times background had their PCR 2 DNA products digested with Fast Digest BshTi and either Fast Digest SalI (Heavy), pfl23II (kappa) or XhoI (Lambda) and ligated (Thermo Scientific) into expression vectors. Ligated DNA was transformed into DH10B chemically competent cells (Intact Genomics, # 1011-24) and grown out on LB agar plates with Carbenicillin (100 mg/L; Teknova C2199). Individual colonies were selected for colony PCR and streaked out on new LB Agar plates with Carbenicillin. EconoTaq Plus green master mix with 0.25 μM AbSense primer and antisense constant primers^43^ were used in PCR with thermocycling conditions of an initial denaturing at 95°C for 5 min, then 30 cycles of 95°C for 30 s, 55°C for 30 s, and 72°C for 60 s, and a final extension at 72° for 5 min before cooling to 12°C. Five μL of PCR products were separated on a 1% agarose TBE gel with SYBR Safe DNA stain. Remaining PCR product was purified using E.Z.N.A Cycle Pure Kit (Omega Biotek; #D6492) for PCR products positive for DNA bands between 500 and 700 bp. DNA underwent Sanger chain-termination sequencing (Genewiz of Azenta Life Sciences, South Plainfield, NJ). Immunoglobulin sequences were analyzed through the use of IgBlast (www.ncbi.nlm.nih.gov/igblast) and IMGT/V-QUEST (http://www.imgt.org/IMGT_vquest/vquest) to determine which sequences should lead to productive immunoglobulin, to identify the germline V(D)J gene segments with the highest identity, and to scrutinize sequence properties. 1301B7, C68.61, SA55, SC27, VYD222 and VIR7229 were previously described^13^^,^^15^^,^^17^^,^^28^^,^^33^^,^^38^ and the heavy and light chain variable regions synthesized by Twist Biosciences (South San Francisco, CA) and cloned into expression vectors in an identical manner as above. Transformed DH10B were grown overnight in Terrific Broth supplemented with 100 mg/L Carbenicillin and plasmid DNA isolated using QIAprep Spin Miniprep Kit (Qiagen #27106). For transfection, 6 million HEK293T cells were seeded in a 10 cm tissue culture dish with DMEM supplemented with 10% Hyclone Fetal Clone II, antibiotics and antimycotic. Plates were incubated for 18 to 24 h overnight at 37°C and 5% CO_2_. Media was exchanged for DMEM supplemented with 5% Hyclone Fetal Clone II, antibiotics and antimycotics. 5 μg of corresponding heavy and light chain plasmid DNA was mixed with 25 μL of jetPRIME® and 500 μL transfection buffer per plate and allowed to incubate for 15 min at room temperature before adding to the plate. Plates were returned to 37°C and media was harvested and replenished 3 times over 8 days. Culture supernatant was concentrated using 100,000 MWCO Vivaspin Turbo 15RC Ultrafiltration centrifugal concentrators (Sartorius, Stonehouse, UK), and IgG captured and eluted from Magne Protein A beads (Promega, Madison, WI) as previously described.^41^^,^^44^ For larger scale antibody production, heavy and light chain variable sequences were provided to GenScript (Piscataway, NJ) for immunoglobulin expression using the TurboCHO platform.

#### Cells and viruses

African green monkey kidney epithelial cells (Vero E6, CRL-1586) were obtained from the American Type Culture Collection (ATCC). A Vero E6 cell line expressing human ACE2 and TMPRSS2 (Vero AT) was obtained from BEI Resources (NR-54970). Cells were maintained in Dulbecco’s modified Eagle medium (DMEM) supplemented with 5% (vol/vol) fetal bovine serum (FBS, VWR) and 1% penicillin−streptomycin−glutamine (PSG) solution (Corning). HEK293T cells were obtained originally from American Type Culture Collection and maintained in DMEM supplemented with 5% FBS (VWR) and antibiotic/antimycotic (Corning, Manassas, VA). SARS-CoV-2 WA.1 strain was obtained from the Centers for Disease Control and Prevention (CDC) through BEI Resources (NR-52281). SARS-CoV-2 XBB1.5 and KP.3 were obtained through BEI Resources (NR-59104 and NR-59892, respectively).

#### Binding characterization

ELISA plates (Nunc MaxiSorp; Thermo Fisher Scientific, Rochester, NY) were coated with recombinant CoV S proteins at 1 μg/mL. Recombinant S proteins used include JN.1 spike, KP3.1 spike, KP3.3 spike, KP3-RBD-2LL-ACE2 (described above), Wuhan-1 spike and SARS-CoV S (BEI Resources). RBD^Wuhan-1^-Fc was expressed in 293Expi cells and purified by protein A affinity chromatography.^33^ Purified mAbs were diluted in PBS, and binding was detected with HRP-conjugated anti-human IgG (Jackson ImmunoResearch, West Grove, PA). Substrate (SureBlue, SeraCare, Millford, MA) was added and after 10 min, the reaction was stopped with 1 N HCl. The OD450 was measured using a Molecular Devices VersaMax plate reader (San Jose, CA).

SPR studies were performed using a Biacore T200. All studies were performed at 25°C in running buffer consisting of 10 mM HEPES, pH 7.4, 150 mM NaCl, 0.0075% P20, and 75 μg/mL bovine serum albumin (BSA). Binding and epitope mapping was performed by capturing RBD^Wuhan-1^-Fc to CM5 sensor chips (Cytiva) using an anti-murine Fc capture kit (Cytiva). Initial binding analysis was performed by injecting 20 nM mAb for 90 s over the RBD^Wuhan-1^-Fc. RU bound was recorded. For the competition assay, two mAbs (each at 50 nM) were sequentially injected (mAb1 followed by mAb2) for 90 s over the RBD-Fc surface with 60 s dissociation phases. The flowrate for the binding and competition studies was 30 μL/min. Binding levels, defined by response units (RU) of each mAb, were recorded following the binding phase of each injection. At the end of each cycle, the surface was regenerated by a 3-min injection of 10 mM glycine pH 1.7. SPR studies using SARS-CoV-2 S trimers were performed using an NTA chip (cytiva) according to manufacturer’s instructions. Following S capture, mAbs were injected over the surface for 120 s, followed by a 120 s dissociation phase at a flow rate of 30 μL/min. SPR kinetic binding data was collected by capturing mAbs to a CM5 sensor chip with the anti-human Fc capture kit (cytiva). RBDs were injected over the captured mAbs for 90 s followed by a 240 s dissociation phase at a flow rate of 50 μL/min. Sensorgrams were fit to a 1:1 model without bulk shift refinement to derive rate constants and *K*D values.

#### Biosafety

All *in vitro* and *in vivo* experiments with live SARS-CoV-2 were conducted in appropriate biosafety level (BSL) 3 and animal BSL3 (ABSL3) laboratories at Texas Biomedical Research Institute. Experiments were approved by the Texas Biomedical Research Institute Biosafety (IBC 20-004) and Animal Care and Use (IACUC 1718 MU) committees.

#### Neutralization assays

Indicated mAbs were tested for neutralization with live SARS-CoV-2 variants as previously described.^45^ Briefly, a mixture of serially diluted mAb (starting concentration of 50 μg/mL) and 200 plaque-forming units (PFU)/well of SARS-CoV-2 in post-infection media were incubated at 37 °C and 5% CO_2_. After 1 h incubation, Vero AT cells (96-well plate format, 4 × 10^4^ cells/well, quadruplicates) were infected with the mAb-virus mixture. After 1 h of viral adsorption, the mixture overlay was changed with 100 μL of post-infection media containing 1% Avicel. At 18 h p.i. (14 h p.i. for SARS-CoV-2 WA-1 strain), infected cells were fixed with 10% neutral formalin for 24 h and immunostained with a mouse mAb (1C7C7) against SARS-CoV nucleocapsid (N) protein that cross-reacts with the N protein of SARS-CoV-2.^45^ Virus neutralization was quantified using an ELISPOT plate reader, and the percentage of infectivity calculated using sigmoidal dose-response curves. The formula to calculate percent viral infection for each concentration is given as [(Average # of plaques from each treated wells–average # of plaques from “no virus” wells)/(average # of plaques from “virus only” wells—average # of plaques from “no virus” wells)] x 100. A non-linear regression curve fit analysis over the dilution curve was performed using GraphPad Prism to calculate NT_50_. Mock-infected cells and infected cells in the absence of mAb were used as internal controls.

The mAbs were also tested using an S-pseudotyped virus (PsV) assay. Pseudotyped SARS-CoV-2 was generated using a protocol established previously^46^ using plasmids available from BEI Resources (NR-53816) to generate pseudotyped virus in HEK293T cells. In short, HEK293T cells were seeded into a 6-well tissue culture plate and the next day when cells reached 50–70% confluency, the S protein expressing plasmid was transfected along with reporter lentiviral backbone (Luciferase-IRES-ZsGreen), and HIV Tat, Gag-Pol, and Rev expressing plasmids using jetPrime transfection reagent (4 μL plus 200 μL buffer per well).To generate PsVs expressing the S protein of JN.1, NB1.8.1 and XFG, sequences were generated by Twist biosciences and used to replace the Wuhan-1 S sequence in the viral entry expression vector. Resulting plasmids were sequenced to ensure correct insertion and were used to transfect HEK293T cells with the other viral packaging plasmids as described above. After three days, cell culture supernatants were collected and clarified by filtration and titration was performed in 96-well plates with 60–80% confluent HEK293T cells expressing human ACE2 (BEI Resources NR-52511) in the presence of 5 μg per mL polybrene (EMD Millipore Corp.). mAbs were serially diluted from an initial concentration (varies for virus and antibody) in 96-well plates, in triplicates. Subsequently, 50 μL of each dilution of mAb was incubated with 50 μL of diluted PsV for 1 h at 37°C, before adding to 60–80% confluent HEK293T cells expressing human ACE2 (again in the presence of 5 μg per mL polybrene). Wells without mAbs (serving as ‘virus alone’ controls) were included in all plates. The plates were then incubated at 37 °C for three days before the quantification of luciferase activity using the Luciferase Assay System (Promega) on a Cytation 3 (BioTek, Winooski, VT). The reduction in luciferase activity for each mAb dose, compared with the ‘virus alone’ controls, was calculated. Neutralization IC_50_ values for the mAbs were obtained by fitting the data to a nonlinear response curve using GraphPad Prism v10.

#### K18 hACE2 transgenic mice experiments

Four to six-week-old female K18 hACE2 transgenic mice were purchased from The Jackson Laboratory and maintained in the animal facility at Texas Biomedical Research Institute under specific pathogen-free conditions and ABSL3 containment. Mice were treated with a single dose of mAb delivered i.p. 6 h prior to viral challenge. Treatments were either 20 mg/kg of 1324A10 or 1332E5, 20 mg/kg of 1324A10 plus 20 mg/kg 1332E5 (high), 10 mg/kg of 1324A10 plus 10 mg/kg 1332E5 (middle), 5 mg/kg of 1324A10 plus 5 mg/kg 1332E5 (low), 40 mg/kg Isotype control IgG1 antibody (Bio X Cell, Lebanon, NH) or PBS. For virus infection, mice were anesthetized following gaseous sedation in an isoflurane chamber and inoculated intranasally with 10^5^ PFU of XBB1.5. For viral titers, mice were humanely euthanized at 2 and 4 d p.i. to collect lungs and nasal turbinate. Tissues from mock or infected animals were homogenized in 1 mL of PBS for 20 s at 7,000 rpm using a Precellys tissue homogenizer (Bertin Instruments). Tissue homogenates were centrifuged at 12,000 × g (4°C) for 5 min, and supernatants were collected and titrated by standard plaque assay in Vero E6 cells and immunostaining. For body weight and survival, four to six-week-old female K18 hACE2 transgenic mice were similarly infected and mice were monitored daily for morbidity (body weight) and mortality (survival rate) for 14 days. Mice showing a loss of more than 25% of their initial body weight were defined as reaching the experimental endpoint and humanely euthanized. K18 hACE2 transgenic mice experiments were conducted once.

#### Cryo-EM analysis

SARS-CoV-2 KP3.1.1 ectodomain containing 6P, furin mutations, and C-terminal his-tags was produced by Genscript. Fabs of 1316C10, 1324A10, 1332D4, and 1332E5 were produced by cleavage of the antibodies with papain for 4 h. Following cleavage, the Fc was removed by protein-A affinity chromatography. The Fabs were concentrated and exchanged into 20 mM HEPES, pH 7.5, 50 mM NaCl and separated from uncut mAb species by gel filtration chromatography.

The cryo-EM samples were prepared by incubating the KP3.1.1 S trimer for 10 min at 37 °C, followed by the addition of a 1.3 molar amount of Fab. The samples (3 μL) were applied to glow discharged C-flat R2/2 400 mesh or Quantifoil R2/1 200 mesh grids in a Vitrobot Mark IV at 0.9 mg/mL concentration, blotted for 4–5 s, and plunge frozen in liquid ethane. Grids were imaged on a Thermo Fisher Glacios 2 microscope operating at 200 kV and outfitted with a Falcon 4i detector using EPU software. All cryo-EM processing, from movies to final refinement, were performed using cryoSPARC^47^ v4.5.3. Masking and density segmentation were performed using segger,^39^ implemented in ChimeraX.^48^ Model building was performed using coot^49^ and isolde.^50^ Real-space refinements were performed using Phenix.^51^ Ribbon diagrams were made using PyMOL.^52^

### Quantification and statistical analysis

Area under the curve (AUC) for ELISA data was determined using GraphPad Prism, v10.0 and values normalized such that the highest value was calculated to be 30 so that a valid comparative heatmap could be generated in GraphPad Prism, v10.0. For viral titers, significance was determined using GraphPad Prism, v10.0. One way analysis of variance (ANOVA) with multiple comparisons test was applied for evaluation of the results between treatments. Significance was declared at *p* < 0.05. For statistical analysis viral titers were log transformed and undetectable virus was set to the limit of detection.
