# Supplementary material for: Emergence of neutralizing RBD antibodies following Omicron infection with limited activity against ancestral SARS-CoV-2
Source: iScience. 2026 Aug 11;29(8):117166. doi: 10.1016/j.isci.2026.117166 (PMC13486884; doi:10.1016/j.isci.2026.117166)
Supplement: Supplementary file 1 [file mmc1.pdf]

iScience, Volume *29*

Supplemental information

Emergence of neutralizing RBD antibodies following Omicron infection with limited activity against ancestral SARS-CoV-2

Michael S. Piepenbrink, Yao Ma, Simran Panjwani, Alexandra R. Blake, Allison M. Bell, James L. Kizziah, Sara H. Mahmoud, Gregory C. Ippolito, Nathan B. Erdmann, Paul A. Goepfert, Luis Martinez-Sobrido, James J. Kobie, and Mark R. Walter

Fig. S1. MAb Epitope Mapping and Specificity Analysis. (**A**) Epitope overlap was determined using an SPR competition assay. The expected binding of each mAb (in RU) to RBD-FC was established with a 50 nM injection of a first mAb. The table reports the fraction binding of a second 50 nM mAb injection (column) following the injection of the first mAb (row). mAbs with completely independent epitopes should have values of 1 (green) and mAbs with completely overlapping epitopes should have values of 0 (red). **(B)** MAbs specificity was evaluated by capturing mAbs to the surface of a CM5 chip using an anti-human capture antibody (Cytiva). RBDs noted in the Figure (20nM) were injected over the mAbs, resulting in the Sensorgrams shown.

A

		inject 1								
		**1316C10**	**1319B10**	**1321D12**	**1314D5**	**1324E3**	**1315A2**	**1324A10**	**1325A4**	**1331H4**
inject 2	**1316C10**	**0.00**								
	**1319B10**	**-0.01**	**0.00**							
	**1321D12**	**0.58**	**0.55**	**0.00**						
	**1314D5**	**0.10**	**0.14**	**0.59**	**0.00**					
	**1324E3**	**0.08**	**0.11**	**0.58**	**-0.01**	**0.00**				
	**1315A2**	**-0.03**	**0.00**	**0.37**	**-0.06**	**-0.05**	**0.00**			
	**1324A10**	**0.03**	**0.07**	**0.52**	**-0.02**	**-0.01**	**0.09**	**0.00**		
	**1325A4**	**0.08**	**0.13**	**0.59**	**0.00**	**0.03**	**0.14**	**0.04**	**0.00**	
	**1331H4**	**0.64**	**0.84**	**0.68**	**0.71**	**0.65**	**0.78**	**0.71**	**0.64**	**0.00**
	**1315H7**	**-0.02**	**0.01**	**0.39**	**-0.05**	**-0.04**	**0.01**	**-0.05**	**-0.06**	**0.78**

B

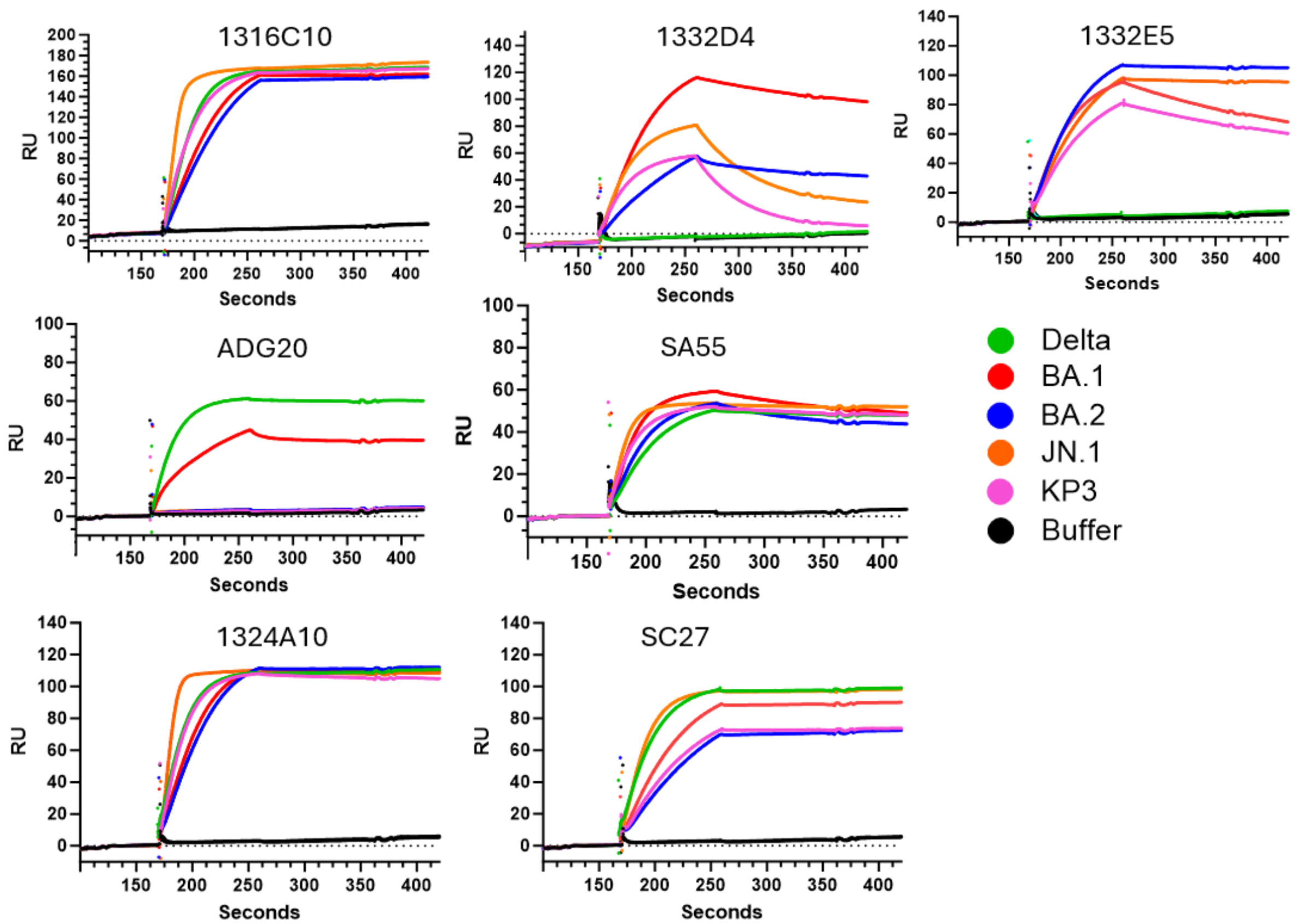

Fig. S2. Neutralization titration curves. MAb titration curves (see Figure legend) against different virus. Datapoints are represented as the mean +/- SD

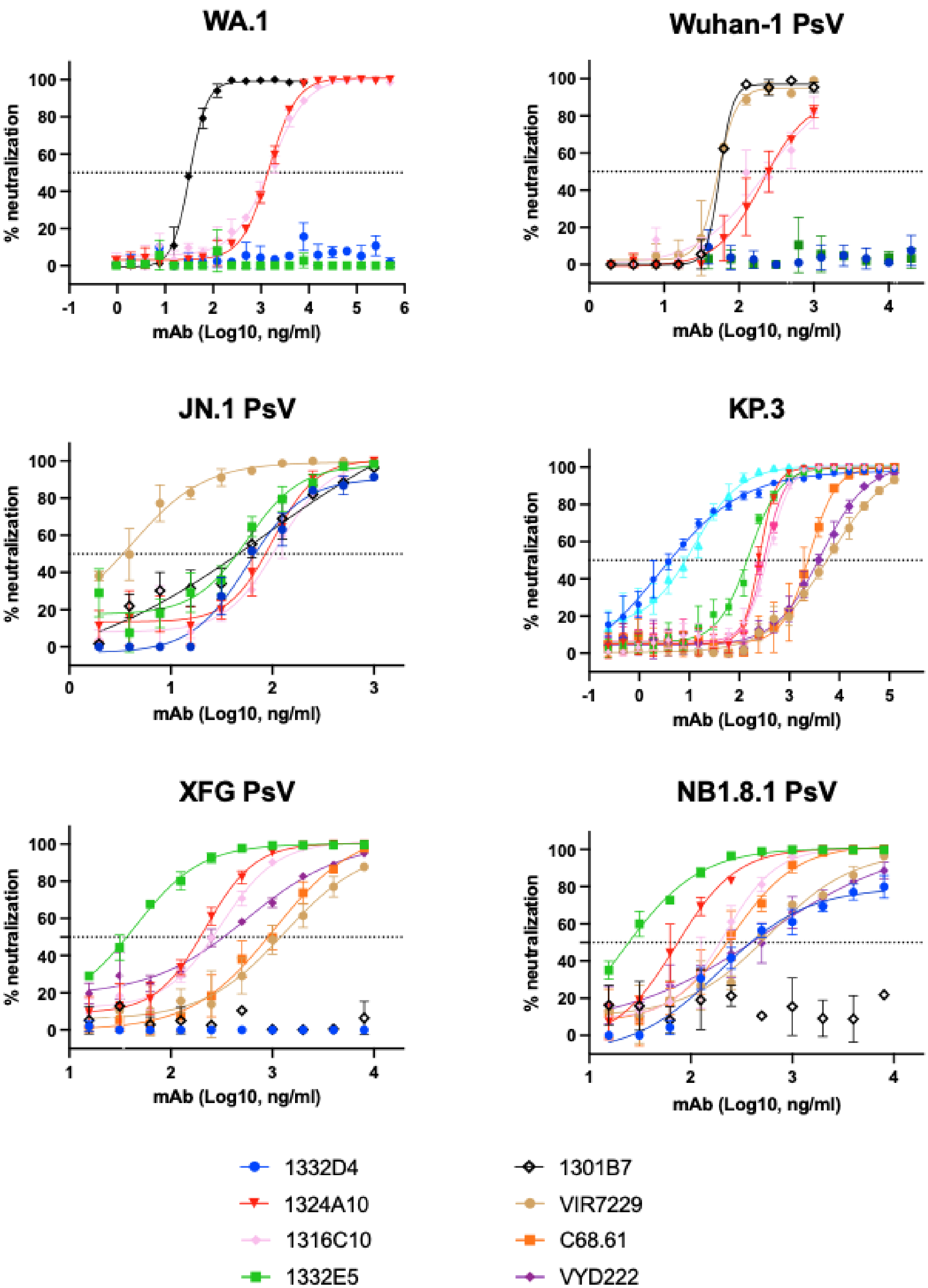

Fig. S3. SPR Sensorgrams and Binding Parameters.

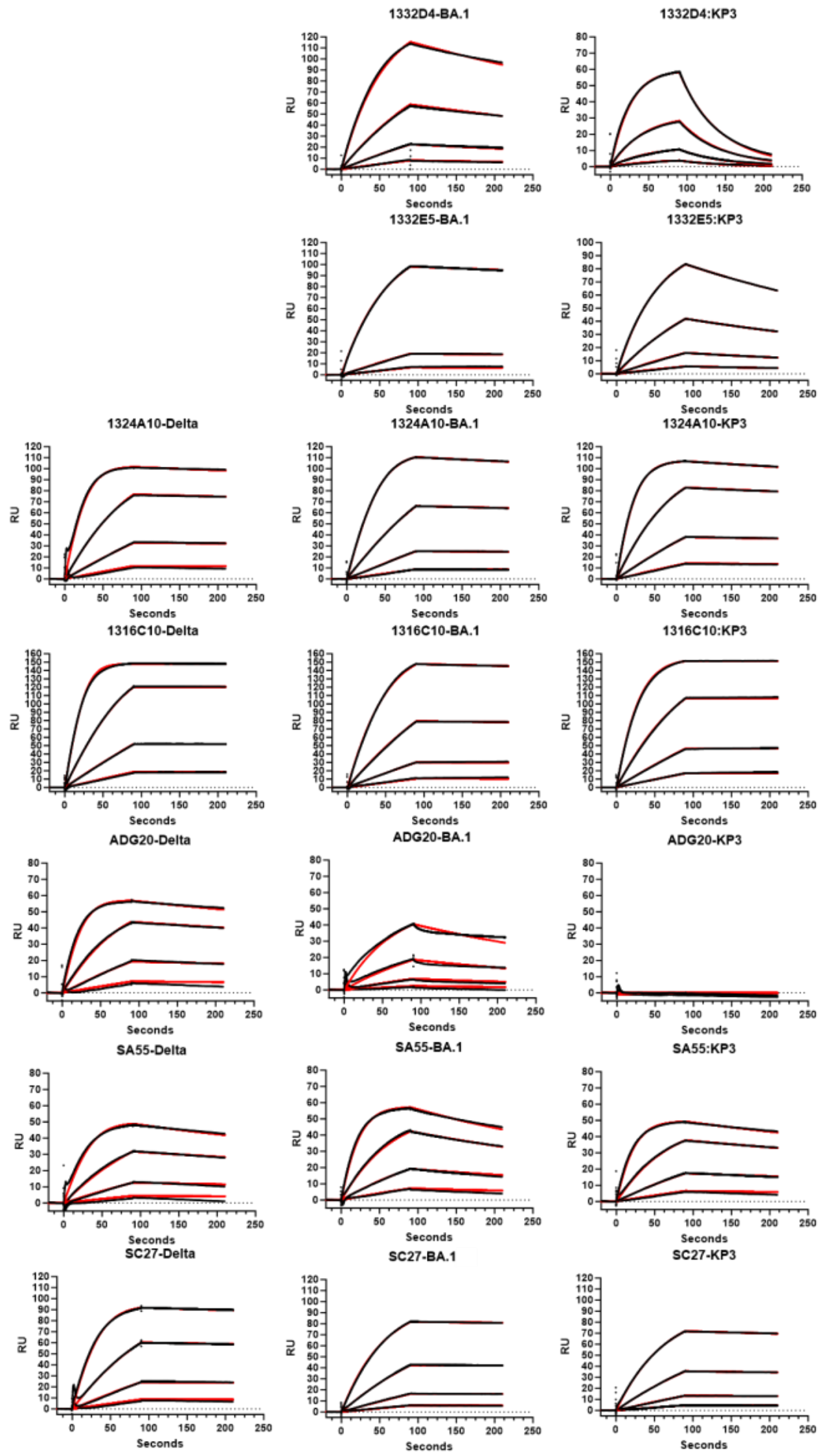

mAb / RBD Binding Parameters				
**mAb**	**RBD**	***ka*** **($M^{-1}s^{-1}$)**	***kd*** **(s^{-1})**	***KD*** **(nM)**
**1332D4**	Delta	-	-	nb
"	BA.1	8.90 (±0.023) x 10^5	1.650 (±0.005) x 10^{-3}	1.85
"	KP3	1.53 (±0.012) x 10^6	2.50 (±0.017) x 10^{-2}	16.34
**1332E5**	Delta	-	-	nb
"	BA.1	8.70 (±0.025) x 10^5	2.78 (±0.043) x 10^{-4}	0.32
"	KP3	9.70 (±0.047) x 10^5	2.41 (±0.006) x 10^{-3}	2.48
**1324A10**	Delta	3.45 (±0.02) x 10^6	2.76 (±0.081) x 10^{-4}	0.08
"	BA.1	2.547 (±0.006) x 10^6	3.49 (±0.019) x 10^{-4}	0.137
"	KP3	3.129 (±0.006) x 10^6	4.21 (±0.026) x 10^{-4}	0.14
**1316C10**	Delta	4.70 (±0.01) x 10^6	*8.6 (±0.023) x 10^{-4}	0.18
"	BA.1B	1.003 (±0.002) x 10^6	8.6 (±0.14) x 10^{-4}	0.9
"	KP3	2.379 (±0.005) x 10^6	*8.6 (±0.026) x 10^{-4}	0.36
**ADG20**	Delta	3.53 (±0.023) x 10^6	8.8 (±0.1) x 10^{-4}	0.2
"	BA.1	6.36 (±0.05) x 105	2.79 (±0.023) x 10^{-3}	4.39
"	KP3	-	-	nb
**SA55**	Delta	2.96 (±0.04) x 10^6	1.42 (±0.018) x 10^{-3}	0.480
"	BA.1	2.87 (±0.013) x 10^6	2.440 (±0.009) x 10^{-3}	0.850
"	KP3	3.03 (±0.013) x 10^6	1.260 (±0.007) x 10^{-3}	0.416
**SC27**	Delta	2.48 (±0.02) x 106	2.23 (±0.083) x 10^{-4}	0.090
"	BA.1	8.68 (±0.075) x 10^5	1.6 (±0.19) x 10^{-4}	0.2
"	KP3	8.8 (±0.1) x 10^5	2.72 (±0.036) x 10^{-4}	0.31

nb = no binding

Figure S4. Data processing of 1332D4-S$^{KP3.1.1}$ cryo-EM data.

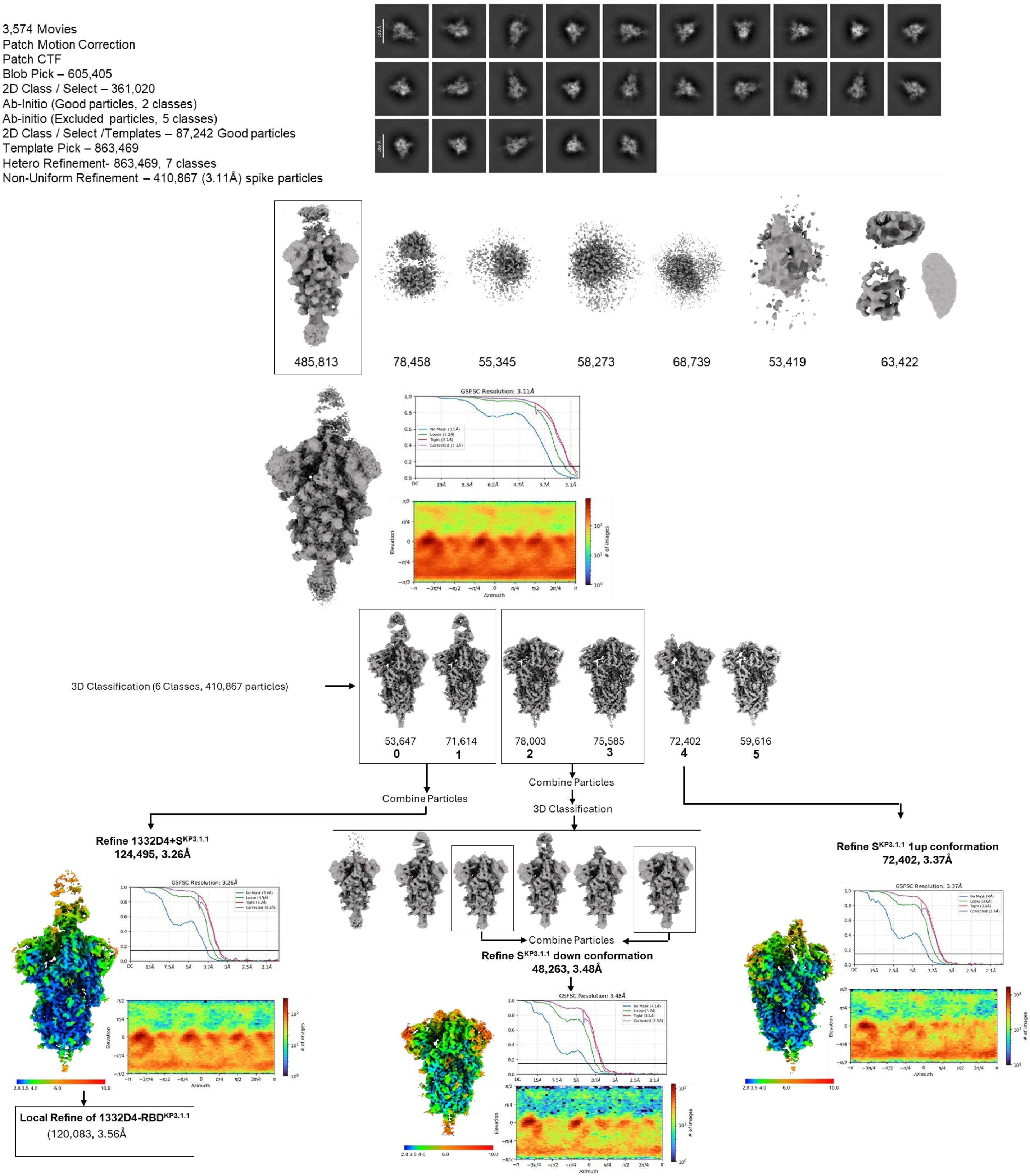

Figure S5. Distribution of S$^{KP3.1.1}$-1332D4 particles and local refinement. Graphical summary of the distribution of particles with Fab (**A**), 1up (**B**) or down (**C**) conformations. Local refinement map, GSFSCs, and view distributions are shown for 1332D4-RBD$^{KP3.1.1}$.

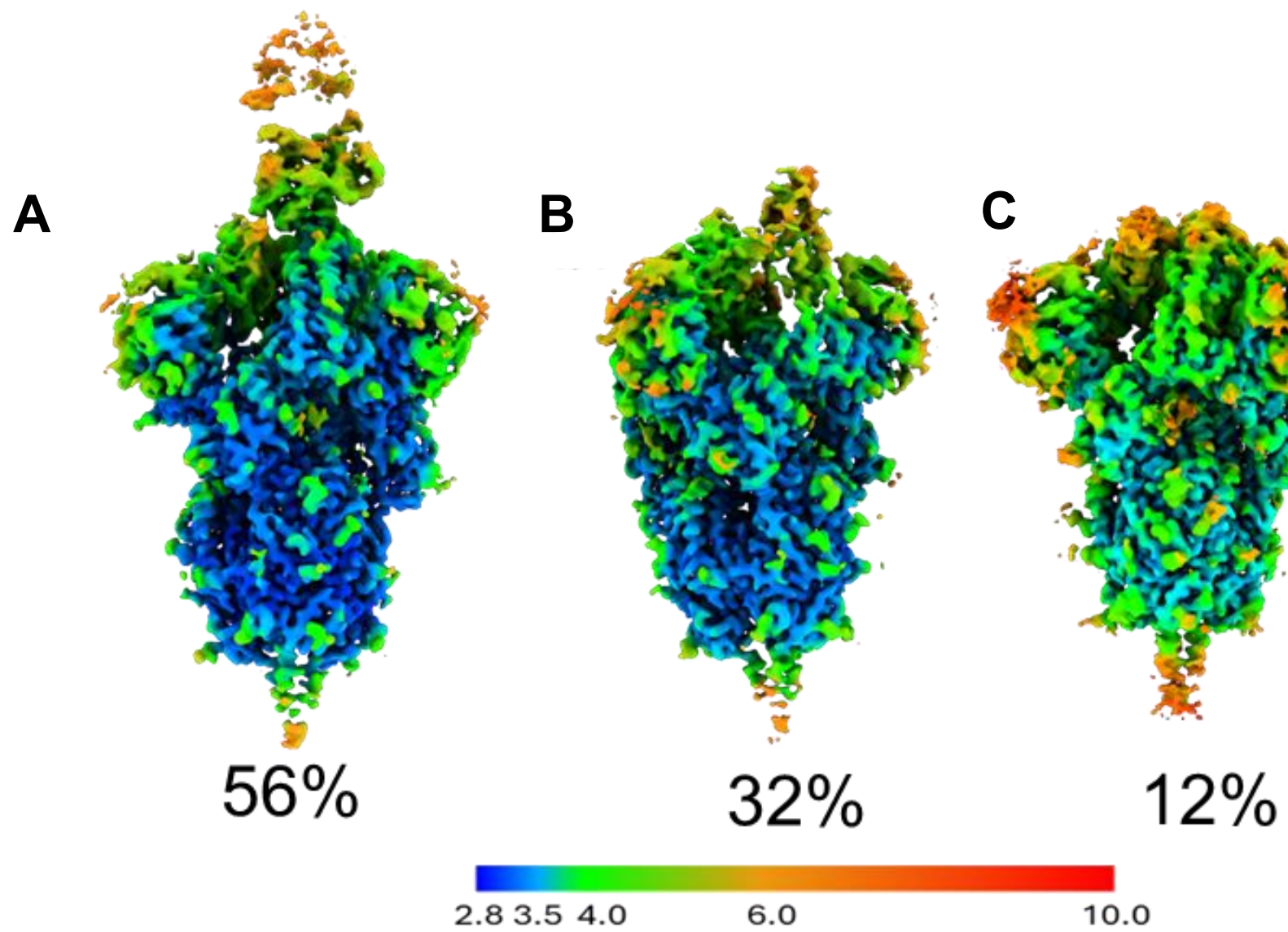

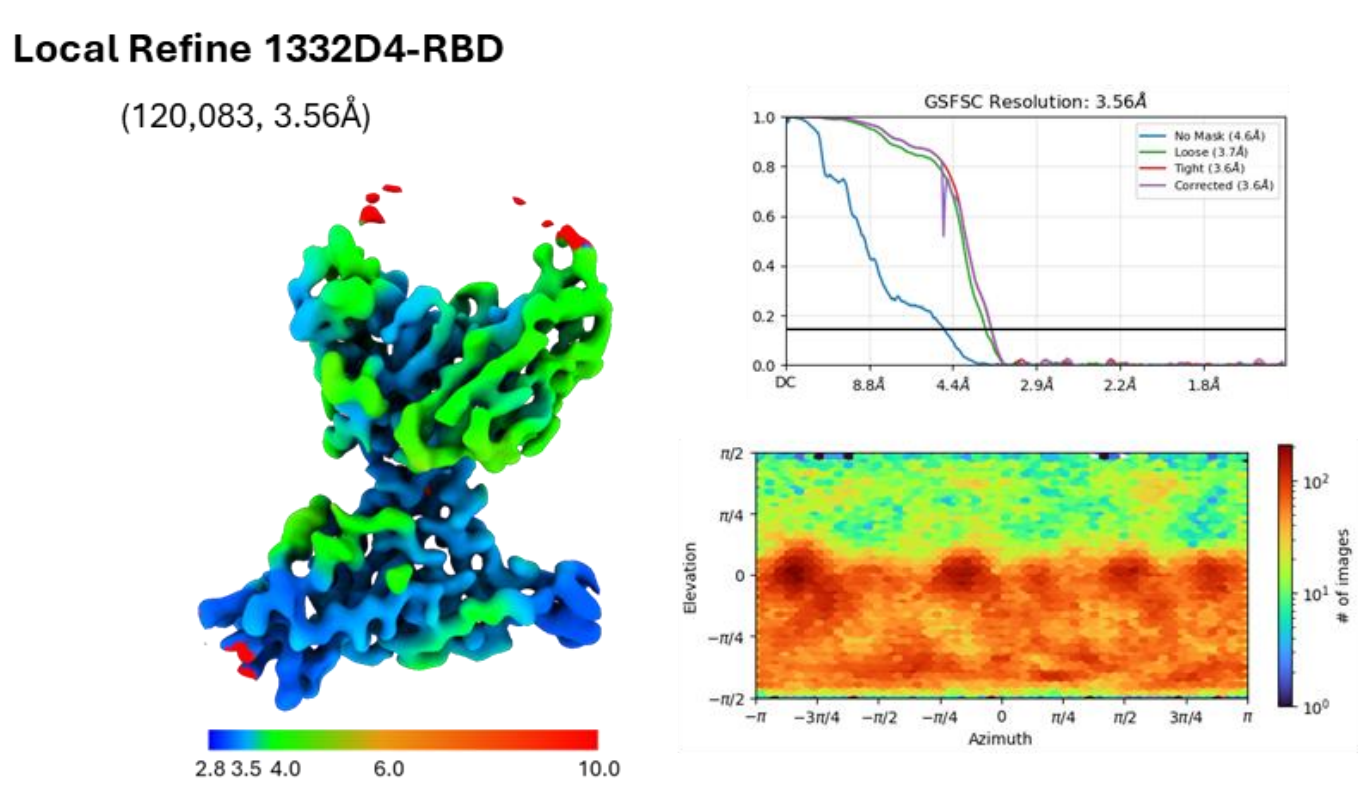

Figure. S6. Data processing of 1332E5-S$^{KP3.1.1}$ cryo-EM data.

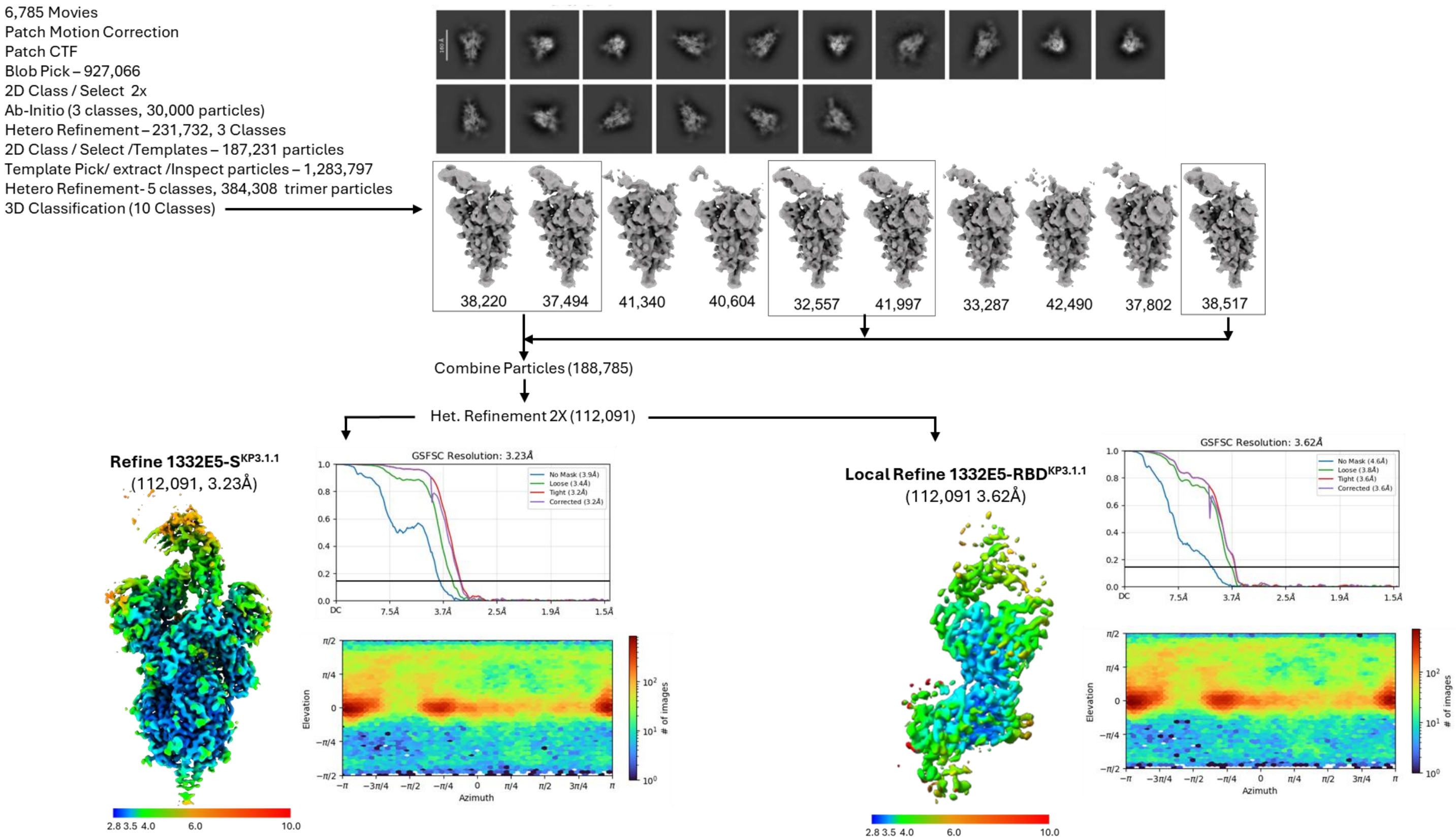

Figure S7. Data processing of 1324A10-S$^{KP3.1.1}$ cryo-EM data.

3,269 Movies
Blob Pick 640,948
2D classification / Select 321,260
Ab-Initio 321,260 particles
Hetero Refinement 6 classes
homo-Refine
3D classification
NU-Refinement
3D classification
NU-Refinement each class

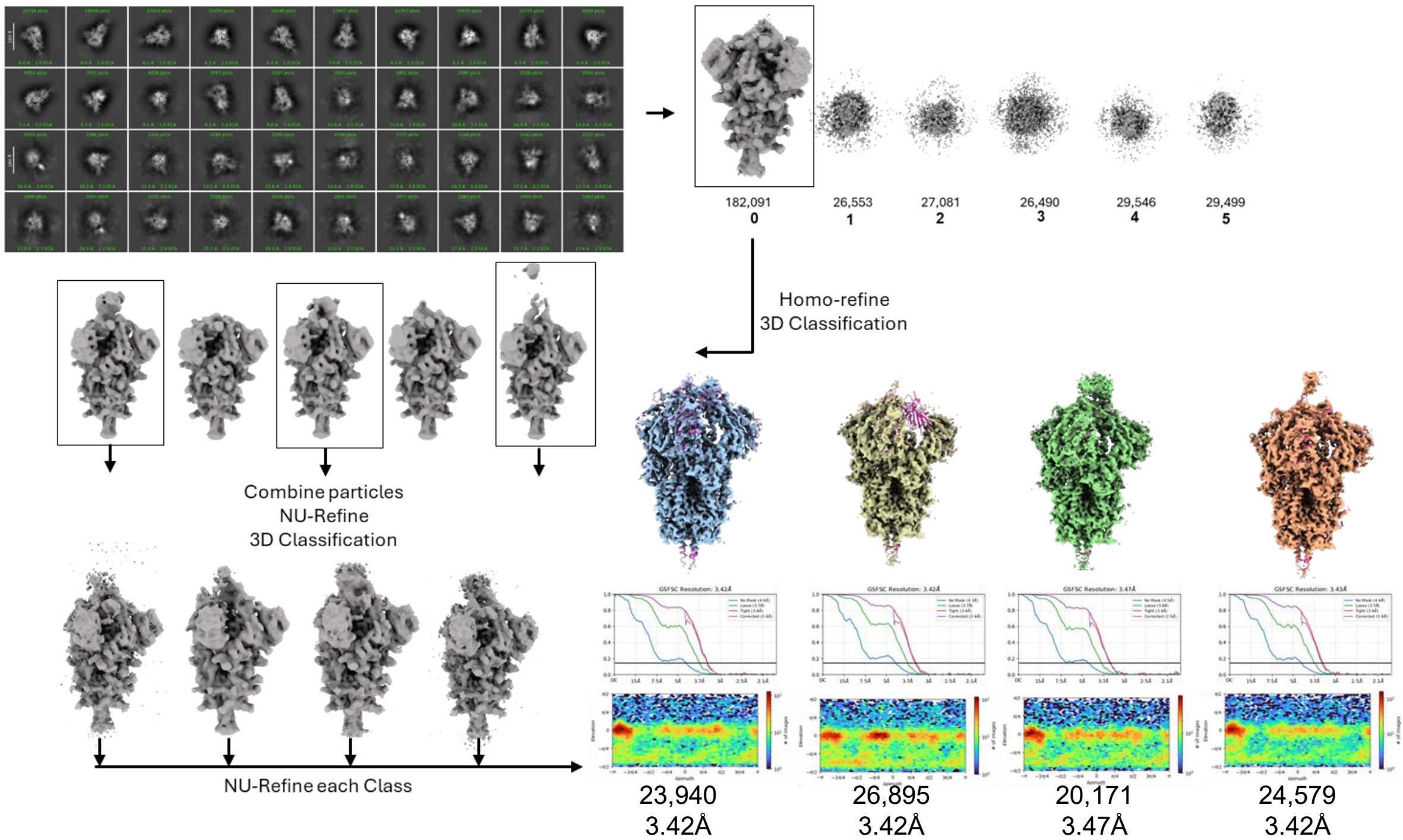

Figure S8. Data processing of 1316C10-S$^{KP3.1.1}$ cryo-EM data.

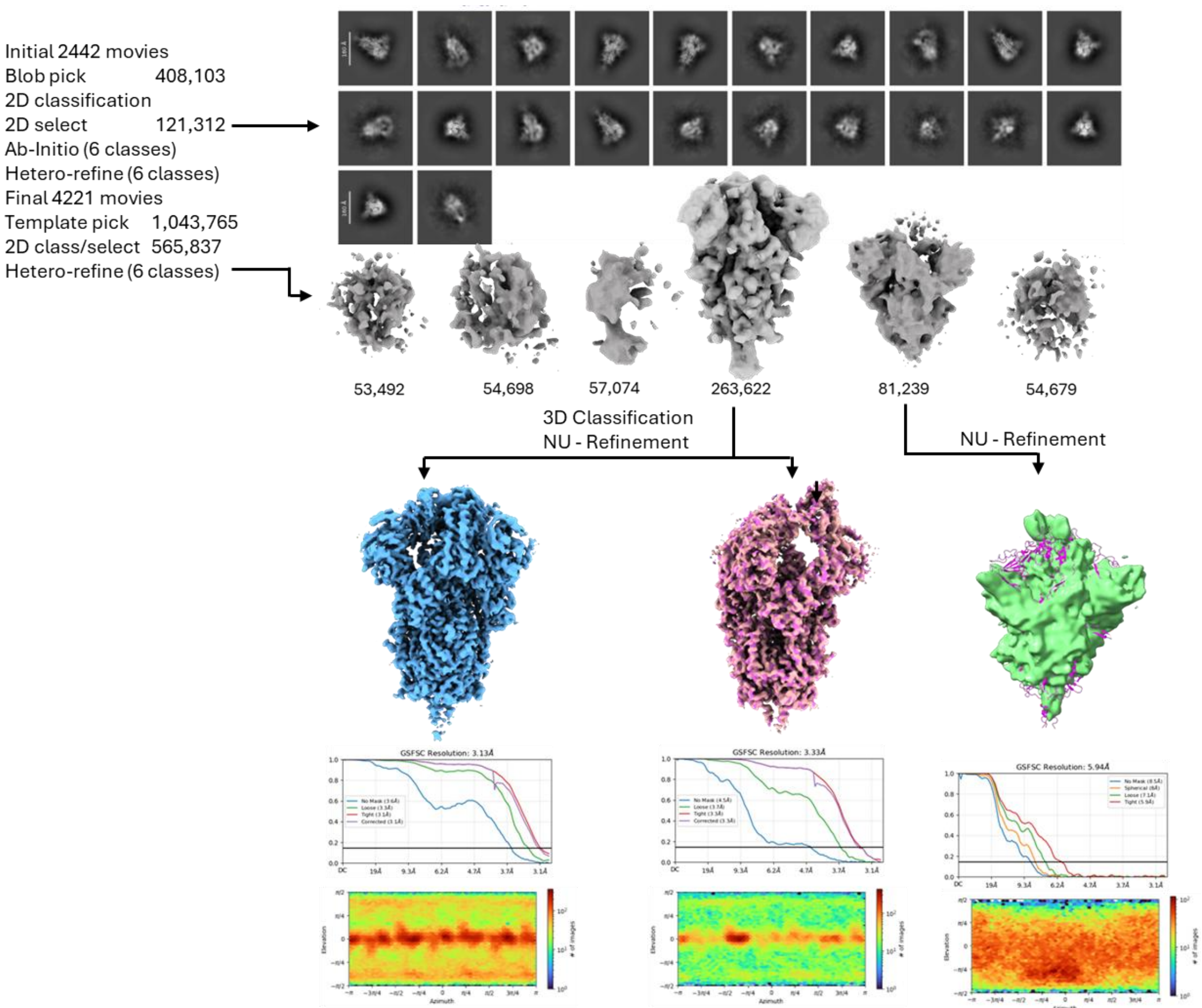

Figure S9. Wuhan-1 PsV Neutralization by Fabs used for cryo-EM studies. Datapoints are represented as the mean +/- SD.

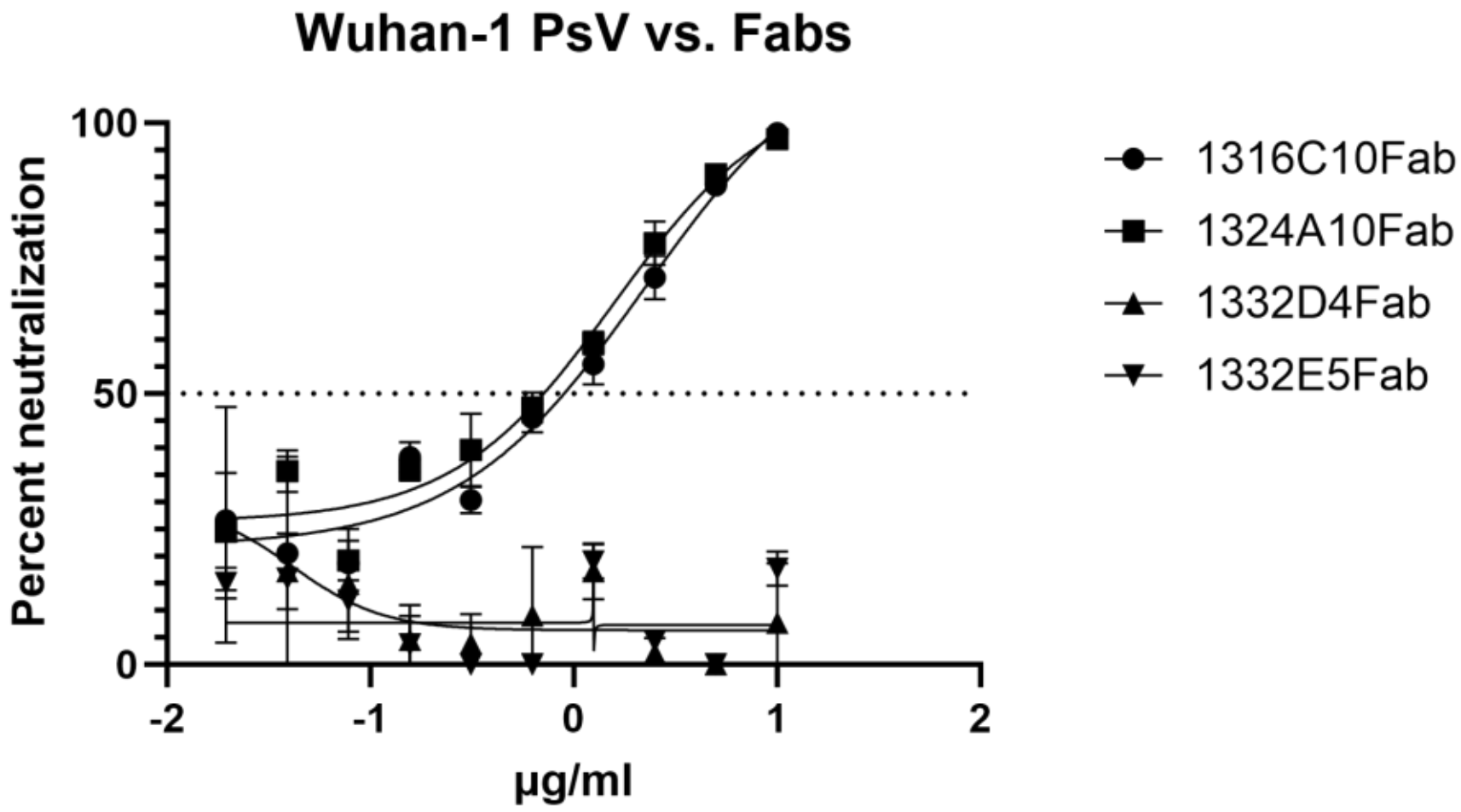

Figure S10. 1332D4 epitope comparison with REGN10987. Ribbon diagrams and molecular surface overlay for the 1332D4 (**A**) and REGN10987 (6XDG) (**B**) epitopes (yellow) on $RBD^{KP3.1.1}$ and $RBD^{Wuhan-1}$, respectively. (**C**) The RBDs were superimposed showing orientations of 1332D4 (orange/blue) and REGN10987 (green). Tables show residue buried surface area for 1332D4 (**D**) and REGN10987 (**E**). Residues colored pink correspond to REGN10987 contacts that are also found in the 1332D4-$RBD^{KP3.1.1}$ complex.

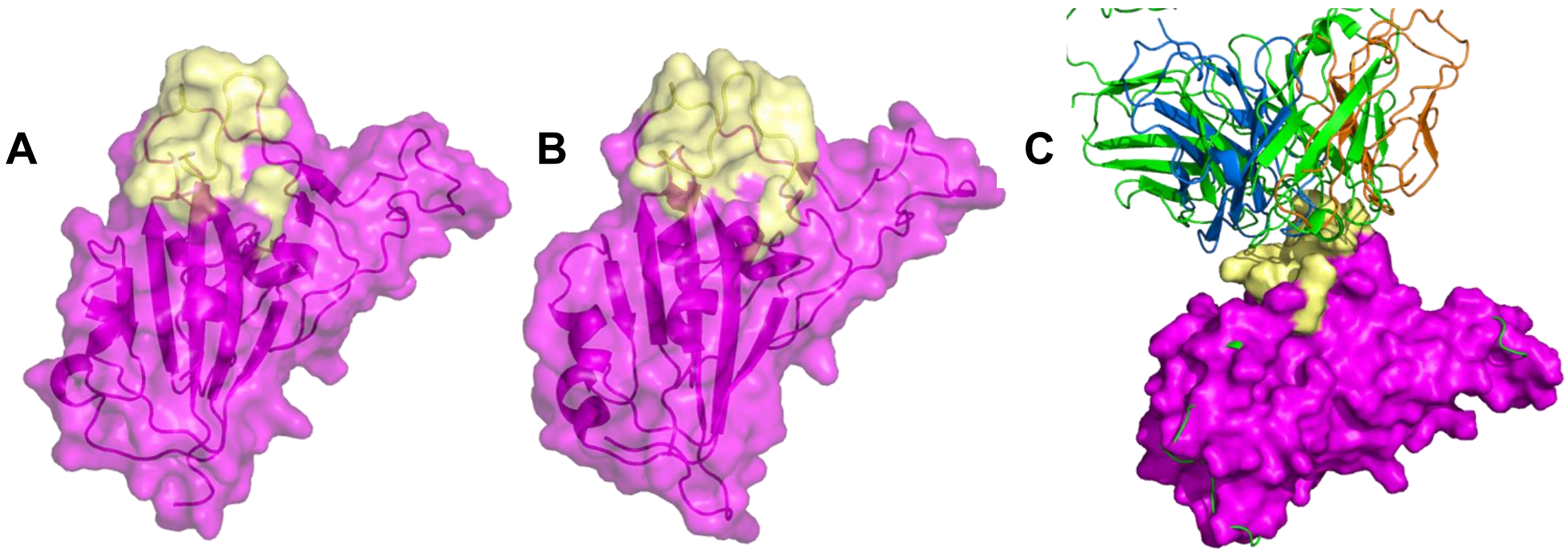

D

RBD-residues	1332D4-HC	1332D4-LC	BSA
B:ARG 346	35.96	35.14	71.1
B:ASN 439	32.23	0	32.23
B:LYS 440	142.59	0	142.59
B:LEU 441	50.52	0	50.52
B:ASP 442	0.83	0	0.83
B:SER 443	11.17	0	11.17
B:LYS 444	67.86	0	67.86
B:HIS 445	115.83	0	115.83
B:SER 446	22.61	45.7	68.31
B:PRO 499	39.66	0	39.66
B:THR 500	13.9	0	13.9
B:TYR 501	4.79	0	4.79
B:GLY 502	0.17	0	0.17
B:VAL 503	3.69	0	3.69
B:GLN 506	14.72	0	14.72
	556.53	80.84	637.37

E

RBD-residues	REGN10987-HC	REGN10987-LC	BSA
E:ARG 346	33.26	0	33.26
E:ASN 439	12.17	0.87	13.04
E:ASN 440	53.48	0	53.48
E:LEU 441	44.97	0	44.97
E:SER 443	11.07	0	11.07
E:LYS 444	79	0	79
E:VAL 445	117.38	18.4	135.78
E:GLY 446	41.55	0	41.55
E:GLY 447	8.11	0	8.11
E:ASN 448	10.46	0	10.46
E:TYR 449	57.01	0	57.01
E:ASN 450	6.34	0	6.34
E:GLN 498	10.85	0	10.85
E:PRO 499	28.05	22.41	50.46
E:THR 500	0	66.01	66.01
E:ASN 501	0	5.35	5.35
	513.7	113.04	626.74

Figure S11. 1332D4 and 1332E5 overlap the RBD-ACE2 binding site. The RBD from the RBD-ACE2 complex (9ELE) was superimposed on RBD of 1332D4-RBD (**A**) and 1332E5-RBD (**B**) complexes. RBD-ACE2 is colored yellow. Heavy and light chains of the Fabs are colored blue and orange, respectively. For the 1332D4-RBD complex, locations of steric clashes are circled in red.

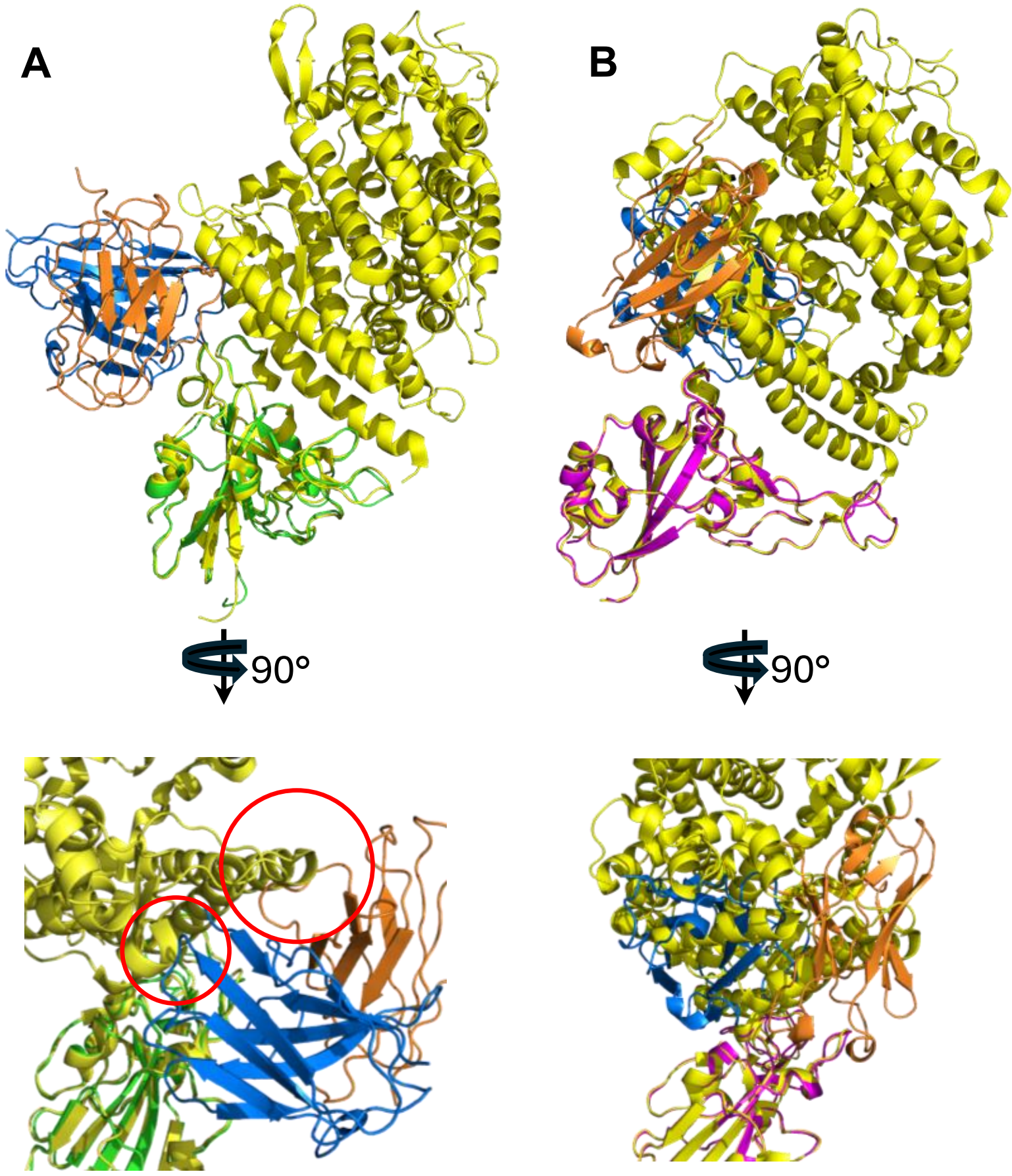

Table S1. Cryo-EM Data Collection and Model Refinement Statistics.

**Data Collection**	**1332D4-S$^{KP3.1.1}$**	**1332D4-RBD**	**1332E5-S$^{KP3.1.1}$**	**1332E5-RBD**
Movies	4,001		6,785	
Movies Accepted	3574*		6,785	
Magnification	190,000		190,000	
Voltage (kV)	200		200	
Dose	50		50	
Defocus range (μm)	1-1.75		1-1.75	
**Map**				
Final number of particles	124,495	120,083	112,091	112,091
Map Resolution Å (FSC 0.143)	3.26	3.56	3.23	3.62
Pixel Size (Å)	0.717	0.717	0.717	0.717
Box Size (voxels)	384	600	512	512
**Refinement Statistics**	Global	Local	Global	Local
Symmetry	C1	C1	C1	C1
Number of atoms	26,776	3,300	26,758	3,257
Number of residues	3,334	413	3,331	419
Number of glycans	51	2	51	0
Mean B, Protein	108.8	56.42	147.9	58.5
Mean B, glycan	126.9	64.62	154.6	85.45
RMS dev. Bond lengths (Å)	0.004	0.003	0.003	0.003
RMS dev. Bond angles (deg.)	0.617	0.562	0.71	0.62
Ramachandran plot				
Favored (%)	96.43	95.58	97.32	95.64
Allowed (%)	3.54	4.42	2.65	4.36
Disallowed (%)	0.03	0	0	0
Ramachandran plot Z-score				
whole	0.12	-2.09	-0.13	-1.23
helix	1.87	-4.13	1.06	-3.94
sheet	-0.01	-1.74	0.28	-0.07
loop	-0.61	-0.84	-0.69	-0.92
Rotamer outliers (%)	1	2.55	1.03	0.56
CaBLAM outliers (%)	2.69	4.24	2.32	2.46
Overall Clash Score	4.69	5.42	5.68	3.61
MolProbity Score	1.48	1.91	1.45	1.45
Correlation Coefficients				
Mask	0.87	0.75	0.87	0.8
Box	0.85	0.53	0.82	0.6
Peaks	0.79	0.49	0.73	0.51
Volume	0.86	0.73	0.86	0.78
ligands	0.77	0.59	0.75	-
**Databases**				
PDB	11HO	11HM	11IS	11IT
EMBD	75698	75696	75726	75725

* rejections if CTF res. > 6Å, Rel. ice thickness <0.94 or >1.06.

Table S2. Comparison of 1332E5 and ADG20 epitopes. Buried surface area (BSA, >5Å^2) for 1332E5-RBD$^{KP3.1.1}$ (left) and ADG20-RBD$^{Wuhan-1}$ (right) calculated using PISA. Residues shared between the mAbs are highlighted in pink. ADG20-RBD data (7U2D).

RBD-residue	1332E5-HC	1332E5-LC	Total BSA
B:PHE 375	25.38	0	25.38
B:GLY 404	7.84	0	7.84
B:ASN 405	75.78	0	75.78
B:ASN 437	0	4.99	4.99
B:ASN 439	0	12.62	12.62
B:LYS 440	0	38.7	38.7
B:HIS 445	0	50.2	50.2
B:ARG 498	15	0	15
B:PRO 499	0	59.44	59.44
B:THR 500	62.89	50.67	113.56
B:TYR 501	44.77	11.11	55.88
B:GLY 502	35.27	7.48	42.75
B:VAL 503	85.27	2.62	87.89
B:GLY 504	27.03	0	27.03
B:HIS 505	82.34	0	82.34
B:GLN 506	0	26.76	26.76
totals>	461.57	264.59	726.16

RBD-residues	ADG20-HC	ADG20-LC	BSA
A:ARG 403	27.40	0.00	27.40
A:ASP 405	59.17	0.00	59.17
A:ARG 408	71.35	0.00	71.35
A:GLN 409	7.42	0.00	7.42
A:ASN 439	0.00	1.17	1.17
A:GLN 498	51.84	0.00	51.84
A:PRO 499	0.00	8.90	8.90
A:THR 500	53.71	66.31	120.02
A:ASN 501	40.97	5.08	46.05
A:GLY 502	26.06	17.59	43.65
A:VAL 503	23.01	80.15	103.16
A:GLY 504	18.27	0.44	18.71
A:TYR 505	107.11	0.00	107.11
A:GLN 506	0.00	23.91	23.91
totals>	486.31	203.55	689.86

RBD-KP3.1.1			Å	1332E3-HC	
B:ASN	405	ND2	3.3	J:TYR 53	OH
B:THR	500	O	3.1	J:ALA 109	N
B:THR	500	O	3.6	J:ASP 110	N
B:THR	500	OG1	3.7	J:GLU 108	OE2
B:GLY	502	N	3.8	J:GLY 107	O
B:VAL	503	N	2.9	J:TYR 50	OH
B:VAL	503	N	3.9	J:TYR 33	OH
B:HIS	505	N	3	J:TYR 33	OH
B:HIS	505	ND1	3.7	J:GLY 107	O
B:HIS	505	NE2	3.6	J:TYR 52	OH
**RBD-KP3.1.1**				**1332E3-HC**	
B:PRO	499	O	2.8	N:SER 96	OG
B:TYR	501	O	3	N:TYR 93	OH
B:GLN	506	NE2	2.9	N:SER 96	O

ADG20-HC	Å	RBD-Wuhan-1
H:SER 56[OG]	3.76	A:ASP 405[O]
H:TYR 33[OH]	2.67	A:ASP 405[OD2]
H:THR 100E[OG1	3.34	A:THR 500[O]
H:GLU 52A[OE2]	2.48	A:ARG 403[NH2]
H:THR 100[O]	3.03	A:GLN 498[NE2]
H:HIS 99[O]	3.21	A:GLN 498[NE2]
H:ALA 100A[O]	2.48	A:THR 500[OG1]
H:ALA 100A[O]	3.65	A:ASN 501[N]
H:THR 100[O]	3.53	A:ASN 501[ND2]
H:THR 100E[OG1	3.75	A:GLY 502[N]
H:ALA 100C[O]	2.76	A:GLY 502[N]
H:GLU 52A[OE2]	2.8	A:TYR 505[OH]
**Salt Bridges**		
H:GLU 52A[OE1]	3.8	A:ARG 403[NH2]
H:GLU 52A[OE2]	2.48	A:ARG 403[NH2]
**ADG20-LC**	**Å**	**RBD-Wuhan-1**
L:TYR 31[OH]	2.94	A:GLN 506[NE2]
L:TYR 91[OH]	3.08	A:VAL 503[N]

Table S3. Primers

Primer name	Sequence (5'-3')
Fragment amplification	
Forward_CMV_Frag[A]	TTCGAGCTCGCCCGACATTGATTA
Reverse_CMV_Frag	AGAATGTACACCGGTTGCAGTTGCT
IgG_Cass_For	CTCC AAG TCG ACC TCTGGGGGCACAGCGGCCCT
Cassette_Rev[B]	AAGGTACCTAACCAAGTTCCTCTTTCAGAGGTTA
Kappa_Cass_For	CGTACGGTGGCTGCACCATCTGTC
Lambda_Cass_For	GTCACTCTGTTCCCGCCCTCGAGTGAGGAG
Heavy (internal PCR-2)	
VH1-Int tag	AGCAACTGCAACCGGTGTACATTCT CAG GTG CAG CTG GTR CAG TCT GGG
VH2-Int tag	AGCAACTGCAACCGGTGTACATTCT CAG RGCACC TTG ARG GAG TCT GGT CC
VH3-Int tag	AGCAACTGCAACCGGTGTACATTCT GAG GTK CAG CTG GTG GAG TCT GGG
VH4-Int tag	AGCAACTGCAACCGGTGTACATTCT CAG GTG CAG CTG CAG GAG TCG G
VH5-Int tag	AGCAACTGCAACCGGTGTACATTCT GAR GTG CAG CTG GTG CAG TCT GGA G
VH6-Int tag	AGCAACTGCAACCGGTGTACATTCT CAG GTA CAG CTG CAG CAG TCA GGT CC
IgG1-Int tag	GTGCCCCCAGAGGTCGACTTGGAGGAGGGTGCCAGGGGGAAGAC
IgG2-Int tag	GTGCCCCCAGAGGTCGACTTGGAGCAGGGCGCCAGGGGGAAGAC
IgA1-Int tag	GTGCCCCCAGAGGTCGACTTGCTGCAGAGGCTCAGCGGGAAGAC
IgA2-Int tag	GTGCCCCCAGAGGTCGACTTGCTGTCGAGGCTCAGCGGGAAGAC
IgM-Int tag	GTGCCCCCAGAGGTCGACTTCTCACAGGAGACGAGGGGGAAAAG
Kappa (Internal PCR-2)	
VK1-Int tag	AGCAACTGCAACCGGTGTACATTCT GAC ATC CAG WTG ACC CAG TCT C
VK2-Int tag	AGCAACTGCAACCGGTGTACATTCT GAT ATT GTG ATG ACC CAG WCT CCA C
VK3-Int tag	AGCAACTGCAACCGGTGTACATTCT GAA ATT GTG TTG ACR CAG TCT CCA
VK4-Int tag	AGCAACTGCAACCGGTGTACATTCT GAC ATC GTG ATG ACC CAG TCT C
VK5-Int tag	AGCAACTGCAACCGGTGTACATTCT GAA ACG ACA CTC ACG CAG TCT C
VK6-Int tag	AGCAACTGCAACCGGTGTACATTCT GAA ATT GTG CTG ACW CAG TCT CCA
VK7-Int tag	AGCAACTGCAACCGGTGTACATTCT GAC ATT GTG CTG ACC CAG TCT
JK1/4-Int tag	GAAGACAGATGGTGCAGCCACCGTACGTTTGATYTCCACCTTGGTC
JK2-Int tag	GAAGACAGATGGTGCAGCCACCGTACGTTTGATCTCCAGCTTGGTC
JK3-Int tag	GAAGACAGATGGTGCAGCCACCGTACGTTTGATATCCACTTTGGTC
JK5-Int tag	GAAGACAGATGGTGCAGCCACCGTACGTTTAATCTCCAGTCGTGTC
Lambda (internal PCR-2)	
VL1-Int tag	AGCAACTGCAACCGGTGTACATTCT CAG TCT GTG YTG ACK CAG CC
VL2-Int tag	AGCAACTGCAACCGGTGTACATTCT CAG TCT GCC CTG ACT CAG CC
VL3-Int tag	AGCAACTGCAACCGGTGTACATTCT TCY TAT GAG CTG ACW CAG CCA C
VL3l-Int tag	AGCAACTGCAACCGGTGTACATTCT TCT TCT GAG CTG ACT CAG GAC CC
VL4ab-Int tag	AGCAACTGCAACCGGTGTACATTCT CAG CYT GTG CTG ACT CAA TC
VL4c-Int tag	AGCAACTGCAACCGGTGTACATTCT CTG CCT GTG CTG ACT CAG C
VL5/9-Int tag	AGCAACTGCAACCGGTGTACATTCT CAG SCT GTG CTG ACT CAG CC
VL6-Int tag	AGCAACTGCAACCGGTGTACATTCT AAT TTT ATG CTG ACT CAG CCC CAC T
VL7/8-Int tag	AGCAACTGCAACCGGTGTACATTCT CAG RCT GTG GTG ACY CAG GAG
VL10-Int tag	AGCAACTGCAACCGGTGTACATTCT CAG GCA GGG CWG ACT CAG
CL-Int tag	CTCCTCACTCGAGGGYGGGAACAGAGTGAC

[A and B] Also used for expression cassette amplification.

[B] Used for amplification of IgG, IgKappa and IgLambda constant fragments.

Table S4. Unadjusted area under the curve ELISA data. Data used to make Table 1C

mAb	CoV Spike	Wuhan-1 Spike	JN.1 Spike	KP3.1 Spike	KP3.3 Spike	KP3-RBD-2LL-ACE2
**1315A2**	5.515	26.91	1.285	14.62	8.866	4.09
**1315H7**	7.266	27	6.006	10.06	7.186	6.4
**1316C10**	6.005	26.89	6.435	5.772	5.08	6.566
**1317D5**	8.069	28.76	4.229	10.68	7.089	8.568
**1319B10**	5.085	26.38	4.207	6.546	7.084	4.935
**1321A9**	6.693	27.54	2.127	13.31	14.73	10.07
**1321D12**	10.94	26.93	1.44	4.88	14.65	10.62
**1324A10**	5.85	27.81	0.8036	12.31	5.051	8.093
**1324E3**	6.321	26.2	0.5769	16.07	7.33	8.871
**1325A4**	3.238	25.62	6.926	12.86	7.493	6.085
**1331H4**	5.406	26.7	7.062	6.596	4.778	6.671
**1332D4**	3.108	0.2481	0.6568	15.55	15.59	3.079
**1332E5**	3.115	2.207	4.512	3.459	5.893	1.674
**SC27**	9.144	24.34	6.566	11.64	9.858	6.016
